# Prophage enhances the ability of deep-sea bacterium *Shewanella psychrophila* WP2 to utilize D-amino acid

**DOI:** 10.1128/spectrum.03263-23

**Published:** 2024-01-03

**Authors:** Xiaoli Tan, Mujie Zhang, Shunzhang Liu, Xiang Xiao, Yu Zhang, Huahua Jian

**Affiliations:** 1 School of Oceanography, Shanghai Jiao Tong University, Shanghai, China; 2 State Key Laboratory of Microbial Metabolism, Joint International Research Laboratory of Metabolic & Development Sciences, School of Life Sciences and Biotechnology, Shanghai Jiao Tong University, Shanghai, China; 3 Yazhou Bay Institute of Deepsea Sci-Tech, Shanghai Jiao Tong University, Sanya, China; 4 Southern Marine Science and Engineering Guangdong Laboratory (Zhuhai), Zhuhai, China; State Key Laboratory of Microbial Resources, Institute of Microbiology, Chinese Academy of Science, Beijing, China

**Keywords:** prophage, deep-sea bacterium, D-amino acid, RDOM, nitrogen metabolism

## Abstract

Prophages are prevalent in the marine bacterial genomes and reshape the physiology and metabolism of their hosts. However, whether and how prophages influence the microbial degradation of D-amino acids (D-AAs), which is one of the widely distributed recalcitrant dissolved organic matters (RDOMs) in the ocean, remain to be explored. In this study, we addressed this issue in a representative marine bacterium, *Shewanella psychrophila* WP2 (WP2), and its integrated prophage SP1. Notably, compared to the WP2 wild-type strain, the SP1 deletion mutant of WP2 (WP2ΔSP1) exhibited a significantly lower D-glutamate (D-Glu) consumption rate and longer lag phase when D-Glu was used as the sole nitrogen source. The subsequent transcriptome analysis identified 1,523 differentially expressed genes involved in diverse cellular processes, especially that multiple genes related to inorganic nitrogen metabolism were highly upregulated. In addition, the dynamic profiles of ammonium, nitrate, and nitrite were distinct between the culture media of WP2 and WP2ΔSP1. Finally, we provide evidence that SP1 conferred a competitive advantage to WP2 when D-Glu was used as the sole nitrogen source and SP1-like phages may be widely distributed in the global ocean. Taken together, these findings offer novel insight into the influences of prophages on host metabolism and RDOM cycling in marine environments.

**IMPORTANCE:**

This work represents the first exploration of the impact of prophages on the D-amino acid (D-AA) metabolism of deep-sea bacteria. By using *S. psychrophila* WP2 and its integrated prophage SP1 as a representative system, we found that SP1 can significantly increase the catabolism rate of WP2 to D-glutamate and produce higher concentrations of ammonium, resulting in faster growth and competitive advantages. Our findings not only deepen our understanding of the interaction between deep-sea prophages and hosts but also provide new insights into the ecological role of prophages in refractory dissolved organic matter and the nitrogen cycle in deep oceans.

## INTRODUCTION

The ocean is the largest ecosystem on earth and contains a great quantity of dissolved organic matter (DOM). More than 95% of the DOM in the ocean is thought to be refractory DOM (RDOM), which can resist microbial utilization and remain undegradable for thousands of years in the ocean interior (1, 2). It is estimated that bacteria are the source of approximately 25% of refractory dissolved organic carbon in the oceans (3). In particular, bacterial peptidoglycan (PG) is an important component of oceanic RDOM (4, 5). Among the diverse RDOM, D-amino acids (D-AAs) are the L-amino acid (L-AA) enantiomer (6), but they are not involved in protein synthesis as L-AAs. Notably, D-alanine (D-Ala) and D-glutamate (D-Glu) are the fundamental components of bacterial PG (7). In addition, other D-amino acids are incorporated into PG, including D-serine (D-Ser), D-aspartate (D-Asp), D-methionine (D-Met), D-tryptophan (D-Trp), and D-phenylalanine (D-Phe) (8–12). Therein, D-Ala exhibits the highest concentrations (2–12 nM) in the marine environment, followed by D-Asp (2–10 nM), D-Glu (1–5 nM), and D-Ser (1–4 nM) (13). In marine sediments, the contribution of D-amino acids to total hydrolysable amino acids increased with increased sediment depth and age, reaching up to 59% (14). Therefore, D-AAs are a potentially important reservoir of carbon and nitrogen for microbes inhabiting the deep sea.

Although D-AAs are normally considered RDOM, they can be utilized by some microorganisms as nutrient sources (4, 15). Recently, many D-AA-utilizing microorganisms have been found in diverse habitats, including soils, limnological waters, marine sediments, and seawater (16–18). For samples from the surface seawater and sediments (depth <356 m) of Kongsfjorden, bacterial strains belonging to 12 families and 3 phyla were enriched in culture with D-AAs as the sole nitrogen source. Moreover, seven D-AA-using bacterial strains were isolated from the enrichment, four of which were affiliated with the genera *Pseudoalteromonas* and *Vibrio* (19). From Sagami Bay sediments (from 800 m to 1,500 m), 28 strains were isolated, most of which belonged to *Alphaproteobacteria* (20). By using sediment from the Mariana Trench, microbes belonging to eight bacterial genera and other unidentified genera were enriched when the sample was supplied with D-AAs as the sole carbon source, and the genus *Halomonas* was dominant in the enriched sample (18). Interestingly, *Nautella* sp. strain A04V, which was derived from deep-sea sediment, robustly grew in medium with D-Val as the sole carbon and nitrogen source, whereas its growth was poor with L-valine (L-Val). In contrast, *Nautella* strains isolated from the shallow sea survived only in medium supplemented with L-Val (20). The metabolism of *Halomonas* sp. LMO_D1, which was isolated from Mariana Trench sediments with a water depth of 8,636 m, was more severely impaired with L-AAs under high hydrostatic pressure compared to their enantiomers (18). Furthermore, the uptake ratio of D-Asp/L-aspartate (L-Asp) by bacterioplankton in the water column increased with depth (the value reached ~1 at 1,000 m), which indicated that the utilization efficiency of D-Asp by mesophyll bacteria was almost as effective as that of L-Asp (21). Collectively, these findings indicated that D-AAs may exhibit high bioavailability for deep-sea bacteria and represent one of the important sources of carbon and nitrogen in benthic environments.

Temperate phages, which can integrate into the bacterial host genome as prophages, are prevalent in the oceans (22). Although it significantly underestimated the true prevalence, a survey of 1,239 publicly available marine bacterial genomes indicated that 18% of these genomes harbored at least one prophage (23). Furthermore, it was suggested that at least half of culturable marine bacteria could produce phage-like particles through chemical induction (24). Notably, multiple lines of evidence have suggested that prophages are ubiquitous in the deep ocean, including deep seawater, hydrothermal vents, and hadal trench sediment (25–27). Temperate phages could regulate the physiological traits of their hosts. For instance, the excision of a temperate tailed-phage P2Sp significantly slowed the growth of its host *Shewanella putrefaciens* W3-18-1 and inhibited the host’s biofilm formation (28). The growth and transcriptome of S. *piezotolerans* WP3 under simulated environmental conditions were significantly impacted by the deep-sea filamentous phage SW1, which is active at low temperature and high pressure (29). Intriguingly, the presence of SW1 also decreased the lateral flagellar synthesis and swarming motility of *S. piezotolerans* WP3 at low temperature (30). Recently, marine prophages have been shown to influence the host bacterium’s lipid and metabolite profiles (31).

It is well recognized that prophages are widely distributed in marine bacterial genomes and actively regulate a variety of physiology and metabolism of their hosts. Meanwhile, marine bacteria, especially those from the deep oceans, can use D-AAs as one of the critical sources of nutrients. However, it remains unclear whether prophages are able to influence the utilization of D-AAs by deep-sea bacteria. In this study, we selected *S. psychrophila* WP2 (hereafter referred to as WP2) and its integrated prophage SP1 as a representative system to test this possibility. As a psychrophilic and piezophilic bacterium, WP2 was isolated from deep-sea sediments of the west Pacific Ocean at a water depth of 1,914 m, and its genome was predicted to contain several prophages (32). Among them, SP1 was predicted to possess an intact genome and exhibited a high excision frequency, indicating that SP1 is an active prophage. In the present study, we first tested the ability of WP2 to utilize the four most abundant D-AAs in marine environments. After determining the effect of prophage SP1 on the consumption of D-Glu and D-Ala by WP2, we then performed transcriptomic analysis, inorganic nitrogen measurement, motility, biofilm formation, and competitive assays to further investigate the relationship between prophage and D-AA utilization.

## RESULTS

### Prophage SP1 influences the utilization of D-AAs by *S. psychrophila* WP2

**Fig 1:**
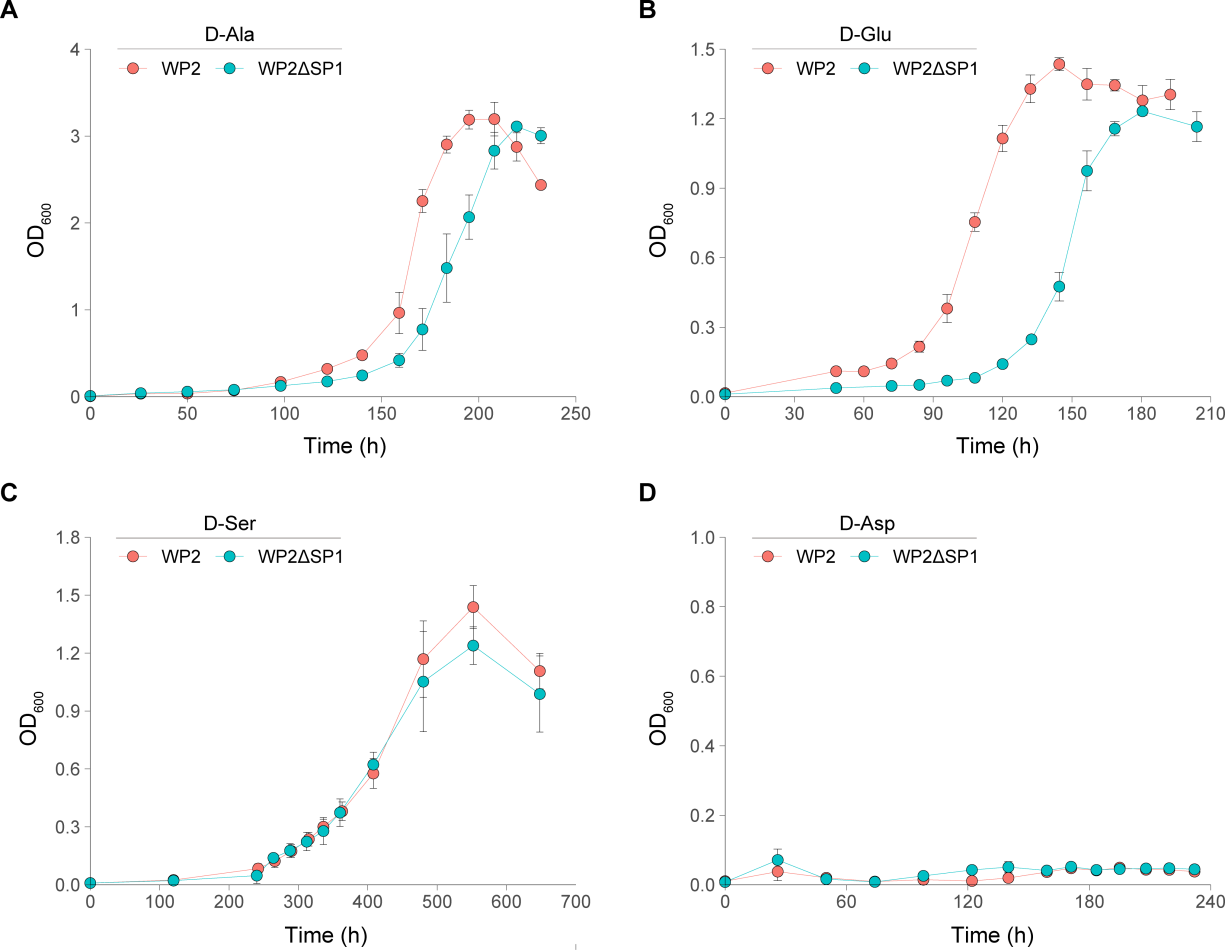
The growth curve of WP2 and WP2ΔSP1 with D-AAs as the sole nitrogen source. Specifically, the strains were cultured in modified LMO-812 medium with (**A**) D-Ala, (**B**) D-Glu, (**C**) D-Ser, and (**D**) D-Asp as the sole nitrogen source. The growth of the strains was detected at an optical density of 600 nm (OD_600_). The data shown represent two independent experiments, and the error bars indicate the standard deviation, which were based on three biologically independent samples.

The genome of WP2 contains genes encoding proline, aspartate, alanine, and glutamate racemases, which can catalyze the enantiomer conversion of these amino acids. In addition, the genome carries D-AA degradation genes, including D-serine deaminase, D-cysteine desulfurase, and D-AA dehydrogenase (Table S1). To determine whether WP2 can utilize the four most concentrated D-AAs (D-Ala, D-Asp, D-Glu, and D-Ser) in seawater, we cultured WP2 at the optimum temperature (15°C) using D-AA as the sole carbon or nitrogen source. The growth assay showed that WP2 could utilize D-Ala, D-Glu, and D-Ser as the sole nitrogen source (Fig. 1) but could not grow when all four tested D-AAs were used as the sole carbon source (Fig. S1).

**Fig 2:**
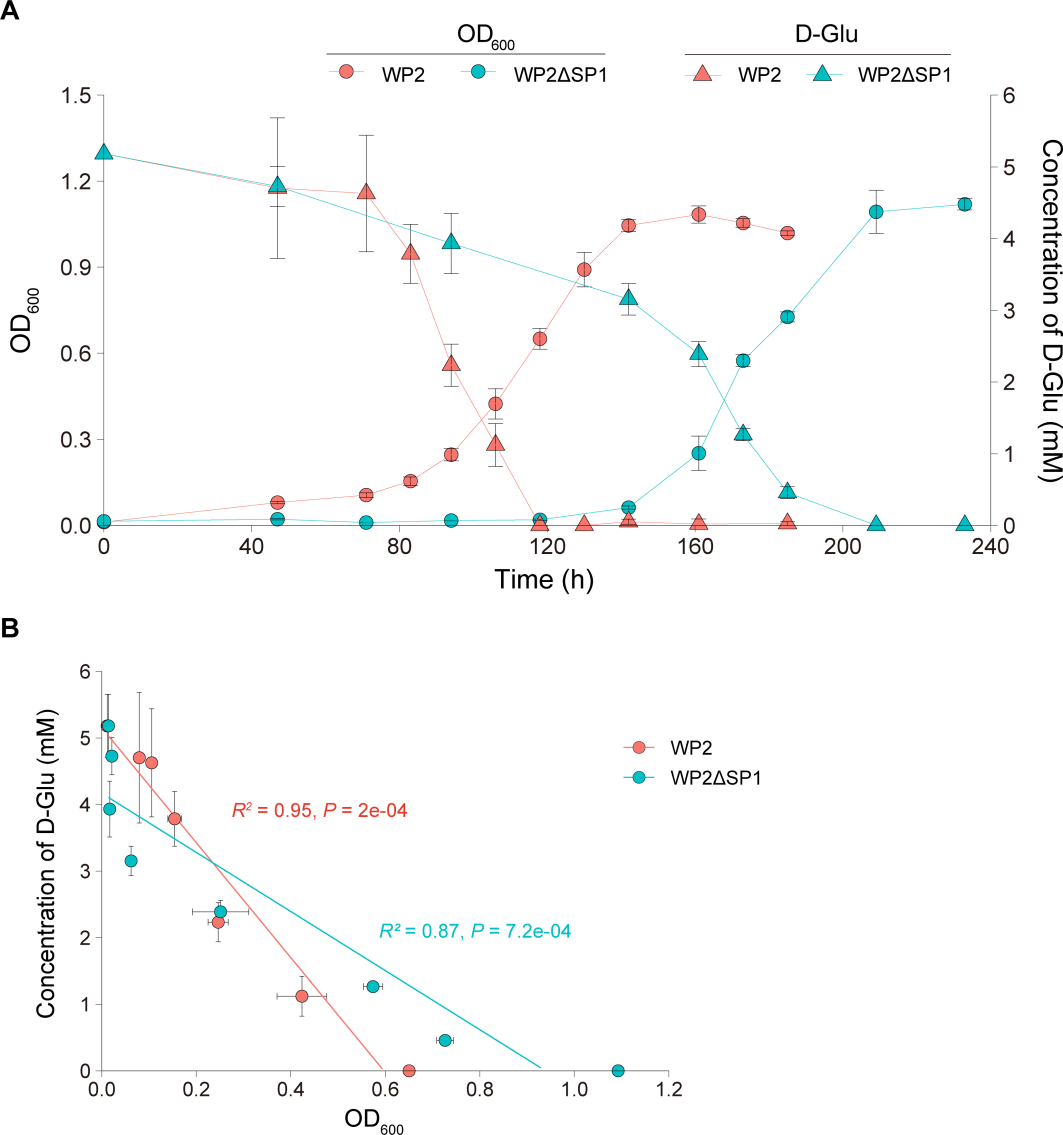
The growth of WP2 and WP2ΔSP1 was significantly correlated with D-Glu degradation. (**A**) The dynamic change in the D-Glu concentration of WP2 and WP2ΔSP1 over the growth phases at 15°C. The strains were cultured in modified LMO-812 medium with D-Glu as the sole nitrogen source. The data shown represent two independent experiments, and the error bars indicate the standard deviation, which were based on three biologically independent samples. (**B**) Correlation analysis between D-Glu concentration and growth of WP2 and WP2ΔSP1. The correlation efficiency (***R*^2^**) and *P* values of linear regressions are shown for each subplot.

To test whether SP1 affects the utilization of D-amino acids by WP2, we examined the growth of WP2ΔSP1, which is an SP1 deletion mutant derived from WP2 wild-type strains. Notably, the growth of WP2ΔSP1 was slower than that of WP2 when D-Glu and D-Ala were used as the sole nitrogen source, and the growth difference between these two strains was more significant in the former than in the latter; in particular, a significantly longer lag phase of WP2ΔSP1 was observed in the D-Glu cultivation (Fig. 1B). We further measured the consumption of D-Glu during the cultivation process, and the results showed that with the growth of the bacteria, the concentration of D-Glu began to decrease until entering the stationary phase when the D-Glu was basically depleted (Fig. 2A), and the growth and the D-Glu concentration were significantly correlated in both WP2 (*R*^2^ = 0.95, *P* = 2E-04) and WP2ΔSP1 (*R*^2^ = 0.87, *P* = 7.2E-04) (Fig. 2B). Notably, the consumption rate of D-Glu by WP2ΔSP1 was significantly slower than that of WP2 in the exponential phase (0.025 vs 0.044 mM·h^−1^, *P* = 1.56E-05), indicating that SP1 significantly affected the rate of D-Glu utilization by WP2. Similarly, the dynamic changes in growth and D-Glu consumption were also significantly correlated (*R*^2^ = 0.98 and *P* = 1.1E-05 for WP2; *R*^2^ = 0.96, *P* = 1.5E-05 for WP2ΔSP1) at 4°C (Fig. S2), and WP2ΔSP1 showed a lower D-Glu consumption rate than that of WP2 (0.014 vs. 0.020 mM·h^−1^, *P* = 1.23E-03), indicating that the influence of D-Glu utilization by SP1 also occurs at low temperature.

To rule out the possibility that mutations in the D-AA metabolism-related genes were introduced during the construction of WP2ΔSP1, we sequenced the genome of WP2ΔSP1, and the genomic comparison showed that it shares an extremely high identity with WP2 [Average nucleotide identity (ANI) = 99.9939%]. Notably, we identified a contig (contig_041, length = 19,350 bp) in the genome sequences of WP2ΔSP1, which covered the region where SP1 was integrated in the WP2 genome, and contig_041 did not have any deletions and mismatches (Fig. S3), indicating that knockout of SP1 does not affect the neighboring genes. We further analyzed single nucleotide polymorphism (SNP) and insertion-deletion (InDel) in the genome of WP2ΔSP1 (Table S2). Compared with WP2, there were 65 SNP sites in the WP2ΔSP1 genome, 49% of which were non-synonymously mutated SNPs (nsSNPs, *n* = 32), and 24 of these nsSNPs were identified in the coding sequence (CDS) region (including 16 genes); Additionally, only 8 of the 40 InDel sites located in the CDS region. Examination of the annotated functions of these genes showed that none of them were involved in the D-AA metabolism of WP2, indicating that the variations in the genome of WP2ΔSP1 compared with WP2 were not responsible for the differences in D-AA degradation between these two strains.

Considering the spontaneous prophage induction and the accompanying lethal effect of the actively released virions that may cause the growth difference between WP2ΔSP1 and WP2, we measured the production of virions in WP2 and WP2ΔSP1 at the early [optical density of 600 nm (OD_600_) = 0.15] and middle (OD_600_ = 0.5) stages of exponential growth phase under the condition of using D-Glu as the sole nitrogen source. The results showed that although WP2 and WP2ΔSP1 can spontaneously produce virus particles (approximately 10^−7^ Virus-like particles (VLPs)/mL) in both stages, there is no significant difference between them (Fig. S4), indicating that the growth advantage of WP2 over WP2ΔSP1 is not due to the spontaneous prophage induction.

### The deletion of SP1 led to significant changes in the transcriptome of WP2 under the growth of D-Glu as the sole nitrogen source

To explore how SP1 affects the metabolism of D-Glu by WP2, we performed transcriptome analysis of WP2 and WP2ΔSP1 when D-Glu was used as the sole nitrogen source. After quality check and read filtration, we obtained a total of 49,198,935 clean reads, which accounted for approximately 89.45% of the total sequencing reads, from six biologically independent samples (Table S3). The clustering and principal component analysis of samples showed that the transcriptomes of WP2 and WP2ΔSP1 were significantly different (Fig. S5). The deletion of SP1 resulted in a total of 1,523 differentially expressed genes (DEGs) in WP2ΔSP1 compared with WP2, of which 680 (excluding SP1 genes) and 797 genes were downregulated and upregulated, respectively (Table S4). To verify the reliability of the transcriptome data, we randomly selected 10 genes for real-time quantitative PCR (RT-qPCR) analysis. The correlation coefficient (*R*^2^) between the RNA-seq and RT-qPCR data was 0.9937 (Fig. S6), indicating that the transcriptome data were reliable and could be used for subsequent analysis.

**Fig 3:**
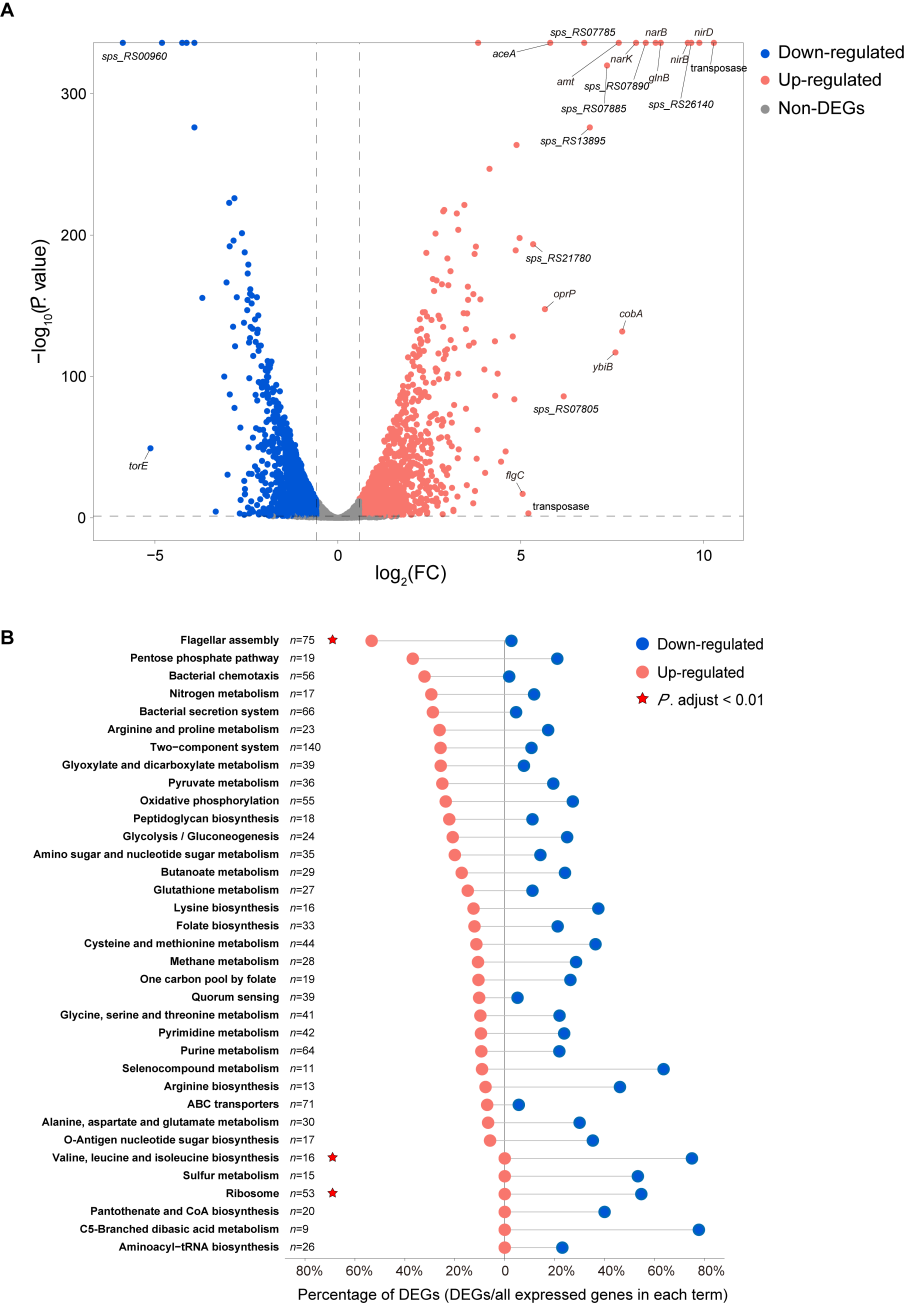
The prophage SP1 significantly influences the transcriptome of WP2 when D-Glu is the sole nitrogen source. (**A**) Volcanic map showing the fold change (FC) of WP2 genes. Each point represents an expressed unigene of WP2 (excluding the genes of SP1). The dashed lines represent the log_2_FC of 1, which was used as the cut-off value for differentially expressed genes (DEGs). The upregulated and downregulated DEGs are indicated in red and blue, respectively. (**B**) Functional category of DEGs according to the KEGG. The percentages of upregulated and downregulated DEGs in each category are indicated as red and blue circles, respectively. The total numbers of genes in each category are shown as *n* = counts. The highly enriched functional categories (*P*.adjust <0.01) are indicated with star remarks.

Since D-Glu is the sole nitrogen source in the culture, we sought to examine the transcription levels of genes related to inorganic nitrogen metabolism in the transcriptome data. Remarkably, the transcription levels of these genes were significantly upregulated in WP2ΔSP1 compared with WP2. Specifically, the expression levels of genes encoding Amt (ammonium transporter), Nark (nitrate/nitrite MFS transporter), NarB (nitrate reductase), NirB (nitrite reductase large subunit), and NirD (nitrite reductase small subunit) were increased at least 32-fold in WP2ΔSP1 (Fig. 3A). Based on the functional category of the Kyoto Encyclopedia of Genes and Genomes (KEGG) database, we performed functional enrichment analysis on DEGs and found that they mainly belonged to eight metabolic pathways (Tables S5 and S6), including flagellar assembly (ko02040), ribosome pathway (ko03010), valine, leucine and isoleucine biosynthesis (ko00290), oxidative phosphorylation (ko00190), C5-branched dibasic acid metabolism (ko00660), selenocompound metabolism (ko00450), cysteine and methionine metabolism (ko00270), and pentose phosphate pathway (ko00030) (Fig. 3B). Among them, flagellar assembly/ribosome and valine/leucine/isoleucine biosynthesis were the most highly upregulated and downregulated (*P*.adjust *<*0.01) pathways, respectively. Moreover, the enrichment scores of genes related to bacterial chemotaxis (ko02030), bacterial secretion system (ko03070), and biofilm formation (ko02025) were all significantly greater than 0 (Tables S4 and S6). Among them, all DEGs related to type VI and III secretion systems were upregulated in WP2ΔSP1, indicating that SP1 inhibited the expression of genes related to these two types of secretion systems. Additionally, the enrichment score value of sulfur metabolism (ko00920)-related genes was remarkably less than 0, and their transcription levels were all downregulated in WP2ΔSP1 (Tables S4 and S6), suggesting that SP1 probably activated sulfur metabolism in WP2. Taken together, these data indicated that SP1 significantly affected the expression of genes related to various metabolic pathways in WP2 when D-Glu was used as the only nitrogen source.

### SP1 affects the dynamic changes in NH_4_^+^ and NO_X_^−^ in WP2

**Fig 4:**
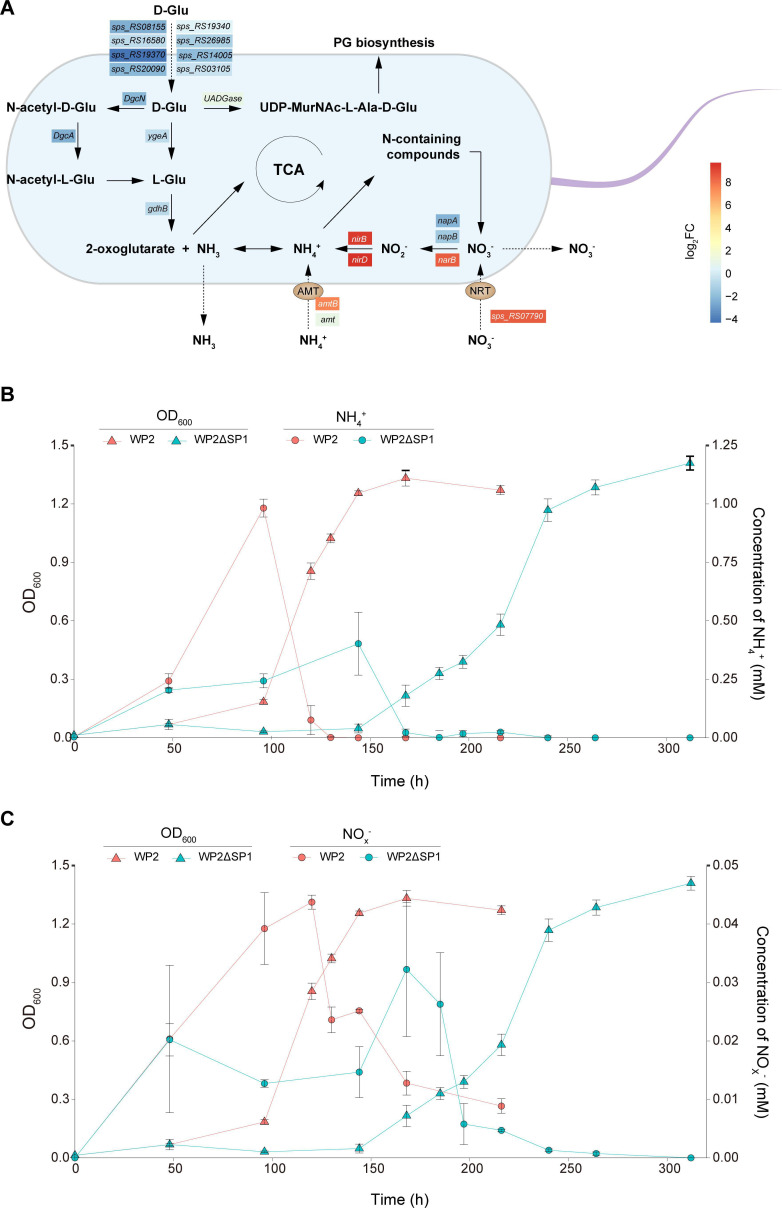
The prophage SP1 significantly influences the extracellular concentration of inorganic nitrogen of WP2 when D-Glu is the sole nitrogen source. (**A**) Schematic diagram displaying the nitrogen metabolism pathway and the related genes in WP2. Normalized fold change (FC) levels of these genes are represented by heatmaps in boxes according to the scale bar (log_2_ scale). (**B and C**) The dynamic change in ammonium (NH_4_^+^) and the total nitrate and nitrite (NO_X_^−^) of WP2 and WP2ΔSP1 over time. The strains were cultured in modified LMO-812 medium with D-Glu as the sole nitrogen source. The data shown represent two independent experiments, and the error bars indicate the standard deviation, which were based on three biologically independent samples.

According to the proposed pathway of WP2 inorganic nitrogen metabolism (Fig. 4A), when D-Glu is the only nitrogen source, WP2 first transfers D-Glu into the cell and converts D-Glu into L-glutamate (L-Glu) through D-Glu racemase (YgeA). Alternatively, D-Glu can be converted to L-Glu through the DgcN-DgcA pathway or directly used for the PG biosynthesis. From the perspective of transcription level [fragments per kilobase per million mapped reads (FPKM) value], the DgcN-DgcA pathway is very likely to be the main pathway for WP2 to utilize D-Glu (Fig. S7). Notably, the transcript levels of *dgcN* and *dgcA* were both significantly downregulated in WP2ΔSP1 compared to WP2 (Fig. 4A; Table S4), which probably resulted in less L-Glu available to WP2ΔSP1, thus leading to a slower growth. In addition, we identified eight genes that may be involved in D-Glu transport from extracellular to intracellular and found that most of them had lower transcript levels in WP2ΔSP1 (Fig. 4A), indicating that WP2ΔSP1 presumably have lower D-Glu transport capacity, and this may also be one of the reasons for its lower growth rate.

After the racemization, the L-Glu is converted to 2-oxoglutarate and NH_3_ by L-Glu dehydrogenase (GdhB). 2-Oxoglutarate can enter the tricarboxylic acid (TCA) cycle, while NH_3_ or its derivative NH_4_^+^, as an important nitrogen source, can form a variety of organic nitrogen compounds. To verify whether SP1 affects growth by affecting the production of NH_3_ by WP2, we measured the concentration of NH_4_^+^ in the medium. The results showed that the concentration of NH_4_^+^ gradually increased during the lag phase and then decreased rapidly with exponential growth of the bacteria (Fig. 4B). Overall, the dynamic change in the extracellular concentration of NH_4_^+^ in WP2 culture was remarkably quicker than that in WP2ΔSP1, suggesting a significantly higher rate of the production and utilization of NH_4_^+^ in WP2, which explains its shorter lag phase and faster growth. Since both the *ygeA* and *gdhB* genes did not show differential expression, why WP2 exhibits a higher NH_4_^+^ production rate remains to be explored in the future. Additionally, the transcription level of two genes encoding the ammonium transporter was significantly upregulated in WP2ΔSP1, corresponding to a lower extracellular NH_4_^+^ concentration in the WP2ΔSP1 cultures.

We further detected the total concentration of nitrate and nitrite (NO_X_^−^) during the cultivation process. The overall concentration of NO_X_^−^ was low (<0.05 mM) but exhibited the same change trend as that of NH_4_^+^ (Fig. 4C). The culture of WP2ΔSP1 showed a steadier change and overall lower concentration of NO_X_^−^ than that in WP2; this result was in accordance with the significantly upregulated transcription levels of NO_X_^−^ transport and reduction genes, including *narK*, *napA*, *napB*, *nirB*, and *nirD* (Fig. 3A; Table S4). Considering the NH_4_^+^ and NO_X_^−^ are crucial components of the nitrogen cycle (33, 34), their dynamic changes resulted from prophage may also have certain impacts on the microbial metabolism and biogeochemical processes in the ocean.

### SP1-induced changes in D-AA utilization by WP2 are accompanied by altered motility and biofilm formation

**Fig 5:**
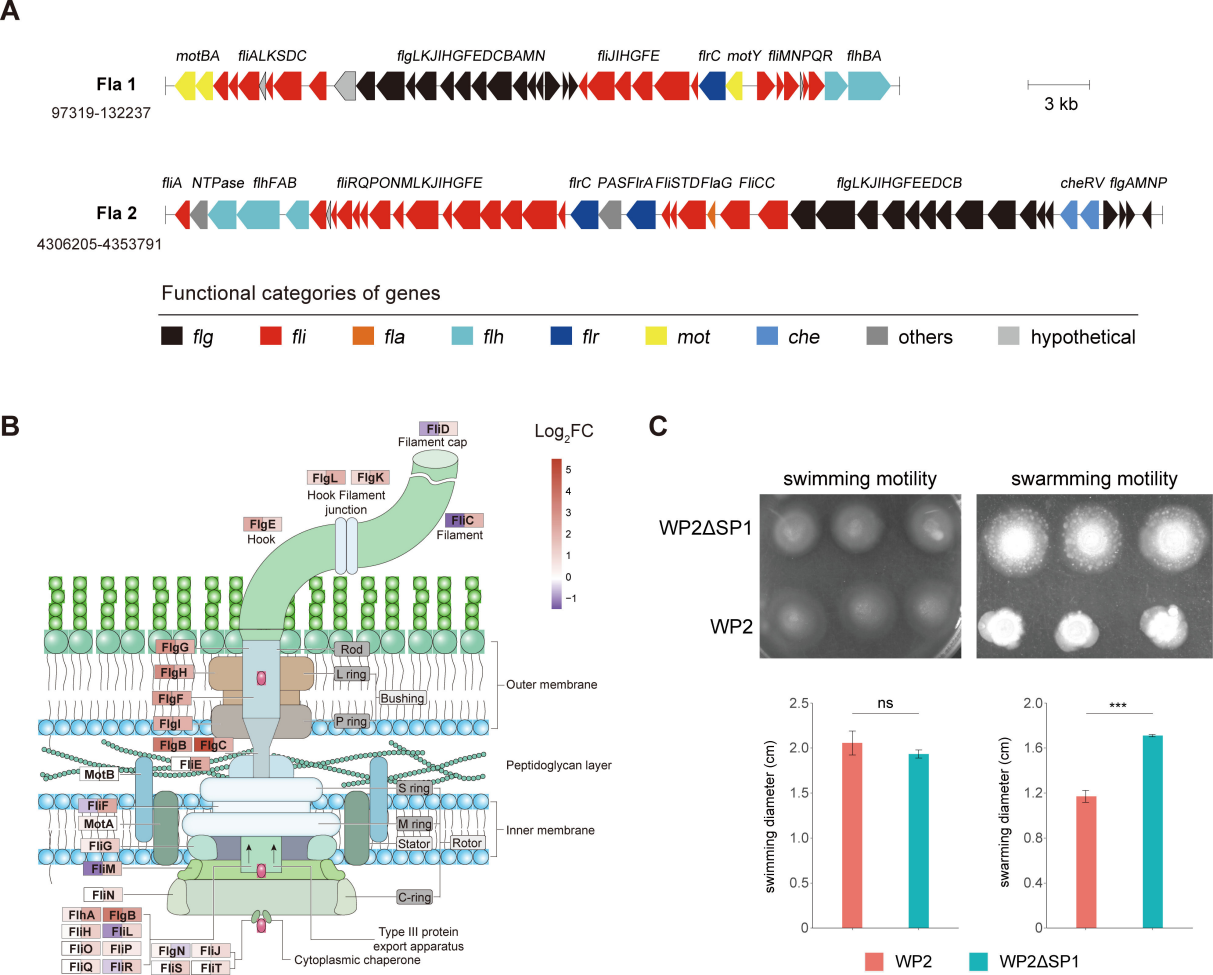
The prophage SP1 significantly decreases flagellar gene expression and swarming motility. (**A**) The flagellar gene clusters of WP2. Different colors are used to represent genes with different functions. Fla 1 and Fla 2 correspond to the lateral and polar flagellar gene clusters, respectively. (**B**) Schematic diagram displaying the differentially expressed flagellar genes and their encoded proteins, which constitute different components of the flagellum. Normalized fold change (FC) levels of these genes are represented by heatmaps in boxes according to the scale bar (log_2_ scale). (**C**) Swimming and swarming motility assays of WP2 and WP2ΔSP1. The data shown represent the results of two independent experiments, and the error bars indicate the standard deviations. The significances were analyzed by two-sided unpaired Student’s *t*-test. ***P < 0.001; ns, not significantly different.

There are two sets of flagella-encoding gene clusters in the WP2 genome, namely, Fla 1 and Fla 2, which encode lateral and polar flagella, respectively (Fig. 5A). Transcriptomic data showed that deletion of SP1 resulted in a significant upregulation of gene expression in both sets of flagellar systems (Table S4). Fla 1 contains 40 genes, among which the DEGs (*flgBCDEFGHIJ*) encode lateral flagellar rod, P/L ring, and hook assembly-related proteins (Fig. 5B). Fla 2 is composed of 47 genes, of which 33 genes are DEGs encoding the MS/C ring, rod, P/L ring, hook, H ring, filament, and stator, which constitute the polar flagellum (Fig. 5B). Considering the significant effect of SP1 on the expression level of flagellar genes, we further performed a motility assay to examine whether SP1 affects WP2 motility. Although knocking out SP1 did not affect the swimming motility of WP2 (1.93 vs 2.06 cm, *P* = 0.203), it significantly enhanced the swarming motility of WP2ΔSP1; as a result, the range of migration of WP2ΔSP1 on the swarming plates was significantly larger than that of WP2 (1.71 vs 1.17 cm, *P* = 6.45E-05) (Fig. 5C). Considering that the synthesis, assembly and operation of the bacterial flagellar system consume a high amount of material and energy (35, 36), the decreased expression of flagellar genes and swarming motility of WP2 compared with WP2ΔSP1 partially explained its faster growth when D-Glu was used as the sole nitrogen source.

Since the motility is tightly correlated to biofilm formation, we then examined the biofilm formation ability of WP2ΔSP1 under the growth condition of D-Glu as the sole nitrogen source at 4°C and 15°C, and the results showed that the deletion of SP1 led to a significant decrease in the biofilm formation of WP2 (Fig. S8). This phenomenon was consistent with previous studies of prophages in other bacteria, including P2Sp in *S. putrefaciens* W3-18-1 (28) and phiv205-1 in *E. coli* (37).

### SP1 conferred a competitive advantage to WP2 when D-Glu was used as the sole nitrogen source

**Fig 6:**
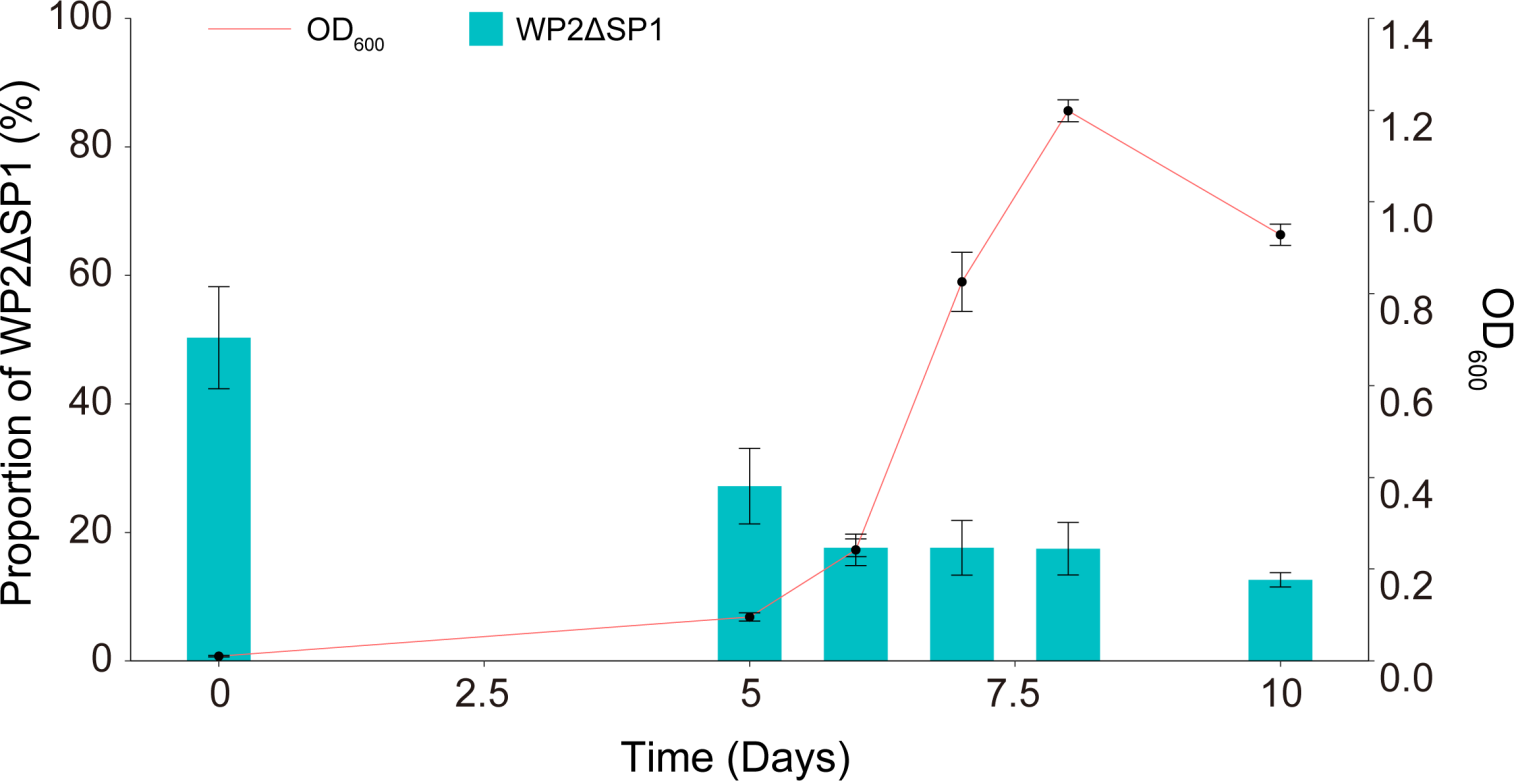
Competition assays of WP2 and WP2ΔSP1. The two strains were cocultured (1:1) in modified LMO-812 medium with D-Glu as the sole nitrogen source. The proportion of WP2ΔSP1 in the culture was determined at different growth phases. The data shown represent two independent experiments, and the error bars indicate the standard deviation, which were based on three biologically independent samples.

Considering that SP1 exerts a positive effect on the utilization of D-AAs of WP2 and that WP2 grew significantly faster than WP2ΔSP1 when they were cultured alone (Fig. 1B), we explored whether SP1 confers competitive advantages on WP2. Therefore, we conducted a competition experiment by coculturing WP2 and WP2ΔSP1 with D-Glu as the only nitrogen source. The examination of the mixed culture at different growth phases showed that the proportion of WP2ΔSP1 gradually decreased with increasing biomass in the culture, and its percentage dropped to approximately 17% after reaching the exponential phase (Fig. 6). This finding confirmed that prophage SP1 endowed WP2 with a competitive advantage when grown with D-Glu as the sole nitrogen source.

### The alteration in the degradation of D-AAs by prophages may occur widely in the ocean

**Fig 7:**
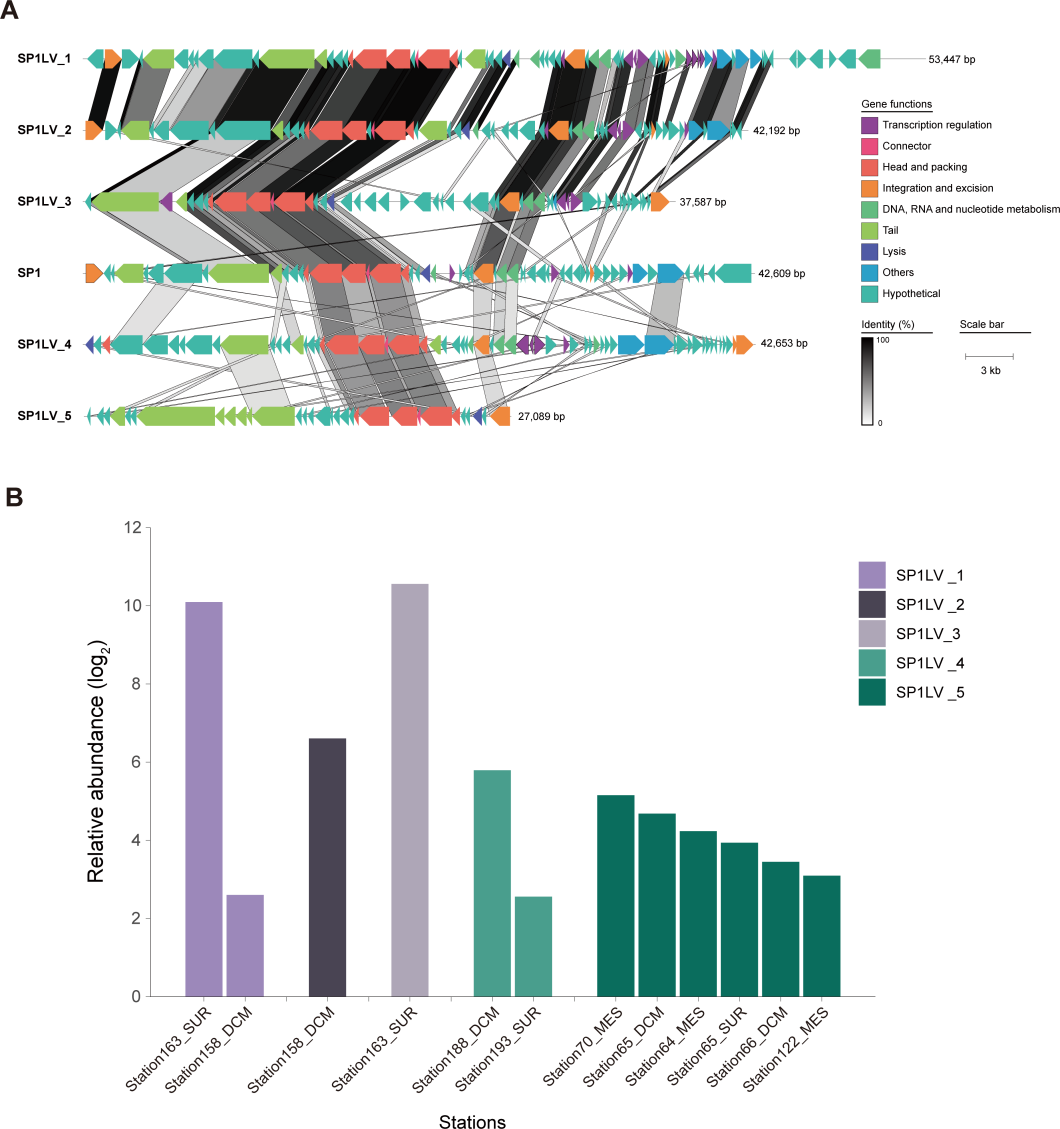
The distribution and relative abundance of SP1-like viruses (SP1LVs) in marine environments. (**A**) Genomic maps depicting SP1-like viruses in the GOV 2.0 data set (38). The arrows depict the location and direction of predicted proteins on the phage genomes, and the filled colors indicate different gene functional categories, as shown in the legend. The protein comparison of SP1 and other viruses was performed by BLASTp alignment with an e-value cut-off of 1e-5. The homologous regions between adjacent genomes are indicated by the shared areas. The bar for percent nucleotide identity is outlined on the right. (**B**) The relative abundance of SP1LVs in the GOV 2.0 data set (38). The relative abundance was calculated as the number of mapped reads per kilobase of SP1LV genomes per billion reads in each virome, and the sampling stations and the relative abundance data were retrieved from iVirus (39). SUR, surface; DCM, deep chlorophyll maximum; MES, mesopelagic.

By measuring the excision frequency of SP1 under different culture conditions, our previous study showed that SP1 is active, and its homologous phages were identified in the genomes of other *Shewanella* strains (unpublished data). To explore the distribution of SP1 in the ocean, we searched GOV2.0, which is the largest marine viral genome data set at present (38). The survey resulted in the identification of five SP1-like viruses (SP1LVs), which have similar gene arrangements and common conserved proteins with SP1 (Fig. 7A). By calculated the average amino acid identity (AAI), these SP1LVs could be grouped into the same family. Specifically, SP1, SP1LV_1, SP1LV_2, and SP1LV_3 were classified in a same genus, and they belong to the same family with SP1LV_4 and SP1LV_5 (Table S7). Relative abundance data indicated that these SP1LVs were widely distributed in different marine environments, including the Arctic Ocean (stations 158, 188, 163, and 193), Indian Ocean (stations 64 and 65), South Atlantic Ocean (stations 66 and 70), and Southern Pacific Ocean (station 122) (Fig. 7B). Moreover, recruitment analysis of SP1 and SP1LVs by Pacific Ocean viromes (POVs) revealed that it is widely present in seawater samples (both shallow and deep) in the Pacific Ocean (Fig. S9). Based on the above evidence, we thus believe that the influence of prophages on the degradation of D-AAs by the host may widely exist in the marine environment.

## DISCUSSION

D-AAs are among the most important components of RDOM, which are widely present in the deep sea and has a higher concentration in marine sediments than in seawater (14). At present, a variety of deep-sea microorganisms that can utilize D-AAs have been isolated (18, 20), and the bioavailability of D-AAs in the deep ocean has been shown to be equal to or even higher than that of L-AAs (20, 40); thus, D-AAs are an important source of nutrients for benthic microbes. In this study, we found that the deep-sea sediment-derived *Shewanella* strain WP2 can utilize D-Glu, D-Ala, and D-Ser as a single nitrogen source for growth. Intriguingly, we found that WP2 possesses a significantly quicker response to D-Glu than that of the prophage deletion mutant WP2ΔSP1. To our knowledge, this is the first report that a prophage can enhance its microbial host to utilize D-AAs. It is worth noting that the impact of SP1 on WP2 growth is not limited to some D-AAs, but probably a more broad-scale metabolic response. To support this claim, we compared the growth of WP2 and WP2ΔSP1 when using other substrates, including L-Glu, L-serine, and NH_4_Cl, as the nitrogen source. The results showed that there was a slightly longer lag phase for the growth of WP2ΔSP1 when L-Glu and NH_4_Cl were used as the sole nitrogen source (Fig. S10), suggesting that SP1 also influences the utilization of other substances by WP2, even though this effect is not as pronounced as that of some D-AAs.

Notably, WP2 can produce a larger amount of extracellular NH_4_^+^, and the highest concentration of NH_4_^+^ in WP2 culture can reach twice that of WP2ΔSP1. In the marine environment, NH_4_^+^ is an important bioavailable nitrogen source and supports the survival of many ecologically important marine microorganisms, including ammonia-oxidizing archaea (41, 42). In addition, we showed that WP2 also produced NO_X_ extracellularly, albeit at a relatively low concentration, during the process of utilizing D-AAs. NO_X_^−^ can also provide important electron acceptors for diverse microorganisms (34). Previously, the *Shewanella* genus was well known for its wide distribution in various marine environments, including the bathypelagic ocean (43, 44). These strains exhibit strong metabolic capabilities and can use a variety of substances as electron acceptors, thus playing an important role in biogeochemical cycles (45, 46). Collectively, our findings contribute novel insight into the ecological function of *Shewanella* in D-AA transformation and the RDOM cycle in the global ocean and indicate that prophages exert a significant influence on the marine nitrogen cycle.

When using D-Glu as the sole nitrogen source, the deletion of SP1 causes a significant impact on the transcriptome of WP2, and approximately 1/3 of the WP2 genes showed significantly differential expression (Table S4). In addition to the inorganic nitrogen metabolism pathway, which was directly related to D-Glu degradation, the DEGs were also associated with a variety of different types of functions (Fig. 3). Interestingly, in previous studies, we found that SP1 showed no significant effect on host growth when the SP1-deleted strain WP2ΔSP1 was cultured in nutrient-rich 2216E medium. Moreover, only 56 DEGs were found in transcriptome analysis [the National Omics Data Encyclopedia (NODE), project ID OEP003432], and these DEGs are distinct from the genes found in the present study, indicating that the influence of deep-sea prophages on the host is dependent on the type and concentration of nutrients in the environment. Overall, when D-Glu is the only nitrogen source, the transcriptome changes caused by the deletion of SP1 first showed that the expression levels of genes related to energy production and protein synthesis were reduced. Specifically, the transcription of the gene cluster atp*ABEFGH* encoding ATP synthase, *nqrABCD* encoding Na(+)-translocating NADH-quinone reductase, and *torADCE* encoding trimethylamine N-oxide reductase was significantly downregulated. In addition, the transcription levels of many genes related to ribosome synthesis and translation, e.g., *rpsO*, *rbfA*, *infB*, *rimP*, *ylpF*, *frr*, *tsf*, *rpsB*, and *arfA*, were also significantly decreased. In contrast, the expression levels of genes related to some energy-consuming systems were significantly upregulated, such as the aforementioned flagellar system and type III and type VI secretion systems. These results indicated that the influence of deep-sea prophage SP1 on the host is multifaceted, and the combined effect of these influences is responsible for the observed differences in growth phenotypes. In addition, these findings suggest that the presence of prophages introduces additional material and energy burdens. As a result, the host must increase energy supply and protein synthesis while reducing some of its own energy-consuming systems, such as the synthesis of the flagellar and secretion systems, thereby maintaining the balance of cell metabolism.

Accompanying the alteration in D-AA utilization, it is very interesting to observe the change in motility in the prophage knockout strain. Flagellar motility has been proposed as an archetypal tradeoff involved in obtaining environmental advantages at the cost of metabolic burden (47). Previously, the effect of prophages on host flagellar gene expression and motility has been widely reported (30, 48–50). In *Escherichia coli*, the deletion of prophage CP4-57 activated the expression of host flagellar gene operons *flg*, *flh*, and *fli*, resulting in approximately eightfold increased motility and reduced early biofilm formation (49). The loss of the deep-sea filamentous phage SW1 promoted the formation of lateral flagella in the host S. *piezotolerans* WP3 and led to an increase in its swarming motility at low temperature (30). Similarly, in this study, we found that the deletion of SP1 led to the upregulation of the expression of lateral flagella genes and an improvement in swarming motility of S. *psychrophila* WP2ΔSP1. Flagella are ecologically important because they play a key role in transporting bacteria toward nutrient-rich environments and away from harmful niches (51, 52), and they are also involved in biofilm formation (53–56). It is conceivable that the influence of prophages on flagella may widely exist and directly affect the adaptability of the microbial host to diverse environments.

Some intriguing questions generated from the current research remain unanswered, especially the underlying molecular mechanism of how SP1 impacts the global transcriptome of WP2 and the detailed pathways and nitrogen flux of WP2 when D-AAs is the sole nitrogen source, which is worthy of further investigation in the future. Although it is possible that novel genes related to nitrogen metabolism are encoded by SP1 (as there are many hypothetical proteins in the SP1 genome, and several of them have relatively high transcript levels), there is currently no evidence to support this speculation. Considering that such a large number of DEGs (*n* = 1,523) were found after SP1 deletion, which is unusual compared with previous reports on other prophages (57–59), it is likely that a pleiotropic regulator encoded by SP1 is involved in the broad-spectrum transcriptional changes of WP2 genes. Specifically, we noticed that SP1 encodes a helix-turn-helix transcriptional regulator (*sps_RS25350*), which has a significantly higher transcription value than other SP1 genes. In fact, several prophage encoding transcriptional regulators have been reported to be able to modulate host gene expression and physiological metabolism. For instance, AppY, a transcriptional regulator encoded by the DLP12 prophage in *E. coli* K-12, increased acid stress resistance and biofilm formation while also caused a strong defect in motility (60). PatE and PsrB, prophage-encoded AraC-like regulators, were involved in transcriptional activation of the acid tolerance pathway in enterohemorrhagic *E. coli* strain EDL933 (61, 62). Recently, the Rac prophage encoded regulator RacR was shown to activate the transcription of a lysozyme encoding gene *lysN*, thus causing the growth defect of *E. coli* strain JM83 (63). While it is possible that the prophage-encoded regulator regulates genes outside of the prophage region, this possibility should be interpreted with cautions. Based on the current understanding of prophage-encoding regulators, they are more likely to specifically regulate the prophage genes, rather than achieving large-scale modulation of bacterial host genes.

It should be noted that compared with WP2, there were 24 nsSNPs and 8 InDel sites in the CDS regions of WP2ΔSP1 genome, and the variation of these genes may be related to the phenotypic changes of WP2ΔSP1. For example, *sps_RS11275* (encoding a methyl-accepting chemotaxis protein), *sps_RS03800*, and *sps_RS28160* (both encoding retention module-containing protein) may be involved in swarming motility (64). In addition, a deletion of one base was found in *sps_RS01795*, which encodes the elongation factor Tu transport aminoacylated tRNAs to the ribosome, and thus has a broad impact on protein biosynthesis (65). Although these genes are not directly responsible for the D-AA degradation and nitrogen metabolism, the possibility that the occurrence of SNP and InDel in these genes affects WP2ΔSP1 transcriptome and other phenotypes cannot be ruled out at present. Moreover, SP1 integrated into the 5′ terminal of *dusA* and *cheX* genes, and the excision or deletion of SP1 probably leads to the alteration of transcriptional control of these two genes (Fig. S3). Among them, the transcription level of *dusA* gene, which encodes a dihydrouridine synthase and has been reported as one of integration hotspots for genomic islands and prophages (66, 67), was significantly downregulated [log_2_ fold change (FC) = −1.879, *P* value = 1.672 E-47] in WP2ΔSP1 compared to WP2. Considering the crucial role of DusA in tRNA modification and its global effect (68), the relationship between decreasing of *dusA* transcription and the large-scale DEGs identified in the WP2ΔSP1 cannot be excluded, but whether the decreased *dusA* transcription involved in the phenotypic changes of WP2ΔSP1 is currently unknown. Additionally, the deletion of SP1 will result in the coding sequence variation of *dusA*, while the alignment of amino acid sequence and protein structure indicated extremely high similarity (identity = 99.4%, TMscore = 98.44) between these two DusA protein variants, suggesting that this variation probably does not influence the function of DusA (Fig. S11).

In this work, we examined the possibility that prophages influence the D-AA metabolism of deep-sea bacteria, with a focus on how prophage SP1 affects WP2’s utilization of D-Glu. We found that SP1 can significantly increase the catabolism rate of WP2 to D-Glu and produce higher concentrations of ammonium, resulting in faster growth and competitive advantages. Transcriptome analysis revealed profound effects of prophage SP1 on WP2 genome-wide transcript levels, suggesting that maintaining material and energy balance could be an important life strategy for the coexistence of deep-sea bacteria and prophages. Despite this progress, overall, our findings not only deepen our understanding of the interaction between deep-sea prophages and hosts but also provide new insights into the role of prophages in RDOM and the nitrogen cycle in deep oceans.

## MATERIALS AND METHODS

### Bacterial cultivation and growth assay

The *Shewanella* strains WP2 and WP2ΔSP1 were incubated in modified LMO-812 medium (69) at 15°C with shaking at 200 rpm. Specifically, the basal components of modified LMO-812 medium contained 26.0 g/L NaCl, 5.0 g/L MgCl_2_·6H_2_O, 1.06 g/L CaCl_2_, 4.0 g/L Na_2_SO_4_, 0.1 g/L KH_2_PO_4_, 0.5 g/L KCl, and 2.52 g/L NaHCO_3_. In addition, the trace element mixture, vitamin mixture (excluding vitamin B12), vitamin B12-only solution, and thiamine solution were added to the medium (1:1,000, vol/vol). When D-AAs (1 g/L) were used as the sole carbon and nitrogen sources, NH_4_Cl (1.60 g/L) and glucose (5.04 g/L) were supplied as nitrogen and carbon sources for the medium, respectively. The pH of the medium was adjusted to 7.0 using 1 M HCl solution. The basal components (excluding NaHCO_3_) were autoclaved, and the remaining components were filter sterilized through 0.22-µm membrane filters (Millipore, USA). For the growth assay, the OD_600_ of the cultures was detected by an ultraviolet spectrophotometer (HACH 6000, Colorado, America) over time.

### Construction of SP1 deletion mutant

The SP1 prophage deletion mutant was constructed by a recombination knock-out method as described previously (58, 70). Briefly, the upstream and downstream fragments flanking both ends of SP1 were amplified, and these two fragments were used as templates in a second fusion PCR, resulting in a fusion fragment flanking the boundary of SP1. Then, the PCR product was cloned into the suicide plasmid pRE112. This plasmid was transformed into *E. coli* WM3064 and then into WP2 by two-parent conjugation. The transconjugant was selected by chloramphenicol resistance and verified by PCR. Afterward, the transconjugant was plated on 2216E agar medium supplemented with 10% sucrose. Finally, the SP1 deletion mutant was screened for and confirmed by PCR and DNA sequencing.

### Genome sequencing of WP2ΔSP1

Cells of WP2ΔSP1 growing in the exponential phase were harvested by centrifugation, and the Genomic DNA was extracted using the Ezup Column Bacteria Genomic DNA Purification Kit (Sangon Biotech, Shanghai, China). The genome was sequenced by Illumina paired-end sequencing technology at Guangdong Magigene Biotechnology Co., Ltd. (Guangzhou, China). We used the sequenced and annotated GenBank file of the genome of *Shewanella psychrophila* WP2 (accession no. CP014782.1) as the reference and mapped the reads of WP2ΔSP1 to WP2 genome. The presence of SNP and InDel in the genome of WP2ΔSP1 was identified by using SAMtools (71) and VarScan (72). The average sequencing depth was 172.33, and regions with sequencing depth ≥100× cover 98.31% of the whole genome.

### Determination of the concentration of D-Glu

The cultures were collected over the growth phases and then filtered using 0.22-µm membrane filters (Millipore, USA), and the supernatants were stored at −20°C. The concentration of D-Glu was determined by a circular dichroism analyzer CD J-1500 (Jasco, Tokyo, Japan) as previously described (73) with slight modifications. Briefly, the scan rate and band width of the instrument were set to 50 nm/min and 1.0 nm, respectively. The spectra were recorded between 200 and 220 nm using 0.1-nm carving, and an average of the three scanning values was taken. The CD spectrum between 205 and 215 nm was chosen for integration, and the integrated value (∑θ) was plotted against the given D-Glu concentration. The fitted standard curve was y (∑θ) = −4,145.9 × (D-Glu concentration) + 16.04 (*R*^2^ = 1).

### RNA isolation and RT-qPCR

The extraction of total RNA was performed using the TRIzol Reagent Kit (Sangon Biotech, Shanghai, China) as previously described (74). Briefly, the crude RNA extraction was treated with DNase I (Thermo Fisher Scientific, Massachusetts, USA) at 37°C for 40 min to remove residual DNA. The purified RNA was reverse transcribed to cDNA using a RevertAid First Strand cDNA Synthesis Kit (Thermo Fisher Scientific, Massachusetts, USA). RT-qPCR was performed in a total volume of 20 µL with PowerUp SYBR Green Master Mix (Thermo Fisher Scientific, Massachusetts, USA) on an Applied Biosystems QuantStudio 3 System (Thermo Fisher Scientific, Massachusetts, USA). The primers used for RT-qPCR (Table S8) were designed using Primer Premier 6.0 (Premier, Canada) software.

### Transcriptomic analysis

Transcriptomic analysis was conducted as previously described (74), and strand-specific transcriptome sequencing was performed at Magigene Biotechnology Co., Ltd. (Guangdong, China). After passing the RNA quality test, ribosomal RNA was removed using the Epicentre Ribo-Zero rRNA Removal Kit (Epicentre, Madison, WI, USA); library construction was performed using the NEBNext Ultra II Directional RNA Library Prep Kit for Illumina (NEB, Ipswich, MA, USA). After library detection was qualified, the Illumina HiSeq sequencing platform (Illumina, San Diego, USA) was used for paired-end sequencing. The raw data were filtered and evaluated with fastp software (75), and the clean reads were then mapped to the *S. psychrophila* WP2 genome using HISAT software (76, 77). RSEM (78) was used to calculate the number of read counts per sample. Then, edgeR was used for differential expression analysis to identify DEGs (79, 80). The identification criteria for DEGs were as follows: false discovery rate ≤0.05 and |log2FC)| ≥1 of the FPKM value between the two strains. The RNA-seq data represent three biologically independent samples for each strain. The functions and pathways of DEGs were enriched by clusterProfiler (81).

### Determining the concentration of inorganic nitrogen

The concentration of inorganic nitrogen in the medium with D-Glu as the sole nitrogen source was determined. The bacterial strains were cultured as mentioned above, and samples were taken at different growth phases. The cultures were filtered through 0.22-µm membrane filters (Millipore, USA), and the supernatant was stored at −20°C. The concentration of ammonium (NH_4_^+^) and the total nitrate and nitrite (NO_X_^−^) were determined using a continuous flow analyzer AA3 (Seal, Norderstedt, Germany) by the standard indophenol blue method and cadmium-copper column reduction method, respectively (82). Since the colorimetric detection method that we used hydrolyze glutamate and, therefore, detect the amine group, the concentration of ammonium (NH_4_^+^) in the medium was calibrated by the equation Y (NH_4_^+^) = 0.0436 × X (D-Glu) + 0.0006.

### Competition assays

Cultures of WP2 and WP2ΔSP1 were grown independently to the exponential phase in modified LMO-812 medium to an OD_600_ of 1.0. A total of 50 mL of each culture was mixed (1:1, vol/vol) and then incubated in LMO-812 medium with D-Glu as the sole nitrogen source at 15°C. The cells were collected by centrifugation over time and stored at −20°C. Then, DNA was extracted by an Ezup Column Bacteria Genomic DNA Purification Kit (Sangon Biotech, Shanghai, China). Rho was used as an internal reference gene, and the primer pair SP1-RT For/Rev flanking the SP1 prophage boundary was used to quantify the proportion of WP2ΔSP1 in the mixed cultures.

### Motility assay

Swimming and swarming motility assays were performed according to a previously reported method (30). In brief, cultures of WP2 and WP2ΔSP1 were grown independently to the early exponential phase (OD_600_ = 0.5) in modified LMO-812 medium. Then, each strain culture was spotted on swimming plates (modified LMO-812 medium with 0.3% agar; Eiken Chemical, Tokyo, Japan) and swarming plates (modified LMO-812 medium with 0.7% agar). For the swimming and swarming motility assays, the plates were incubated at 15°C for 10 days and 15 days, respectively. The motility was assessed by measuring the migration distance of bacteria from one side of the colony edge to the other (maximal swimming and swarming distance).

### Biofilm assay

The biofilm formation assay was performed as previously described (83) with some modifications. Briefly, WP2 and WP2ΔSP1 strains were grown to the early exponential phase (OD_600_ = 0.5) in modified LMO-812 medium with D-Glu as the sole nitrogen source. Then, 200-µL bacterial culture was transferred into 96-well polystyrene plates and incubated for 2 days at 4°C and 15°C, respectively. Afterwards, the supernatant was discarded, and the plates were washed with PBS buffer and fixed with methanol for 15 min. After drying, 1% (wt/vol) crystal violet were used for staining and then washed with ultrapure water. Finally, 95% (wt/vol) ethanol was added in the plates, and the absorbance of the solution was measured at 595 nm.

### Quantification of VLPs

The VLP quantification was performed as previously described (84). In brief, approximately 2 mL of bacterial culture was centrifuged, and the supernatant was filtered with a 0.02-µm pore-size Anodisc Al_2_O_3_ filter (Whatman, Maidstone, England). The filter was stained with 25× SYBR Gold (Invitrogen, Carlsbad, CA, USA) for 15 min in the dark. After rinsing with 0.02-µm filter-autoclaved MilliQ H_2_O, each filter was mounted on a glass slide with 0.1% (vol/vol) p-phenylenediamine dihydrochloride anti-fade mounting medium (Sangon Biotech, Shanghai, China). VLPs on the filter were observed and enumerated with a fluorescence microscope (Olympus BX63, Tokyo, Japan).

### Identification, taxonomic classification, and abundance evaluation of SP1LVs

The viral genome sequences in Global Ocean Viromes 2.0 (GOV 2.0, *n* = 488,131) (38) were downloaded from iVirus (39). The Open reading frames (ORFs) of viruses from GOV 2.0 and SP1 were predicted by Prodigal (v2.6.3) (85) using the parameter “-p meta.” Protein sequences of SP1 were aligned to the viral proteins in GOV 2.0 by BLASTp using Diamond (v2.0.2.140) (86) with identity and e-value cut-offs of 30% and 1e-5, respectively. Only viruses with a length longer than 20 kb and more than five homologous proteins were considered SP1LVs. Genome comparison of SP1 and SP1LVs was performed using Clinker (v0.0.23) (87), and viral gene function categories were assigned by aligning viral proteins to the PHROG database (88) by BLASTp using Diamond (v2.0.2.140) (86) with identity and e-value cut-offs of 30% and 1e-5, respectively. The relative abundance of SP1LVs in GOV 2.0 was retrieved from iVirus (39). The taxonomic classification of SP1LVs at the genus and family ranks was performed by amino acid alignments as previously described (89). As supplement, vConTACT2 (v2.0) was also used for the generation of viral clusters, which approximately correspond to viral genera (90). The read recruitment of SP1LVs in the POV (91) was performed by BLASTn as previously reported (92), with an e-value cut-off of ≤10^−3^.

### Comparison of DusA protein variants of WP2

Multiple alignment of the amino acid sequences the N-terminal of DusA proteins was performed by MEGA X (v11.0.13) (93). The pairwise AAI between DusA proteins was calculated by by BLASTp using Diamond (v2.1.7.161) (94). The 3D structures of DusA proteins were modeled by ColabFold (v1.5.2) (95) with default parameters, and the structure of DusA protein variants of WP2 was visualized by PyMol (v2.0) (96). The structural similarity comparison of DusA proteins was evaluated based on the TM-score, which was calculated by Foldseek (v8.ef4e960) (97).

## Data Availability

The transcriptomic data and the genome sequence (including the raw reads) of WP2ΔSP1 from the current study have been deposited in the National Omics Data Encyclopedia (NODE) data under the project IDs OEP003838 and OEP004233, respectively.

## SUPPLEMENTAL MATERIAL

Supplemental figures and tables spectrum.03263-23-s0001.pdf Supplementary Figures S1-S11; Supplementary Tables S1, S3, S7, S8,

Table S2 spectrum.03263-23-s0002.xlsx Supplemental table.

Table S4 spectrum.03263-23-s0003.xlsx Supplemental table.

Table S5 spectrum.03263-23-s0004.xlsx Supplemental table.

Table S6 spectrum.03263-23-s0005.xlsx Supplemental table.

ASM does not own the copyrights to Supplemental Material that may be linked to, or accessed through, an article. The authors have granted ASM a non-exclusive, world-wide license to publish the Supplemental Material files. Please contact the corresponding author directly for reuse.
