# Supplementary material for: Prophage enhances the ability of deep-sea bacterium Shewanella psychrophila WP2 to utilize D-amino acid
Source: Microbiol Spectr. 2024 Jan 3;12(2):e03263-23. doi: 10.1128/spectrum.03263-23 (PMC10845958; doi:10.1128/spectrum.03263-23)
Supplement: Supplemental figures and tables — Supplementary Figures S1-S11; Supplementary Tables S1, S3, S7, S8, [file spectrum.03263-23-s0001.pdf]

Supplementary Information

Prophage enhances the D-amino acids utilization of deep-sea bacterium *Shewanella psychrophila* WP2

Xiaoli Tan[1#], Mujie Zhang[2,3#], Shunzhang Liu[2], Xiang Xiao[2,3,4], Yu Zhang[1*], Huahua Jian[2,3*]

[1]School of oceanography, Shanghai Jiao Tong University, Shanghai, China.

[2]State Key Laboratory of Microbial Metabolism, Joint International Research Laboratory of Metabolic & Development Sciences, School of Life Sciences and Biotechnology, Shanghai Jiao Tong University, Shanghai, China

[3]Yazhou Bay Institute of Deepsea Sci-Tech, Shanghai Jiao Tong University, Sanya, China

[4]Southern Marine Science and Engineering Guangdong Laboratory (Zhuhai), Zhuhai, China

[#]These authors contributed equally to this work.

*Correspondence: jiandy@sjtu.edu.cn (H.J.); zhang.yusjtu@sjtu.edu.cn (Y.Z.)

Supplementary Figures S1-S11

Supplementary Tables S1-S8

Table S1. The genes related to the D-amino acids metabolism in *S. psychrophila* WP2 genome.

Table S2. Genomic variants in WP2ΔSP1 by compared with WP2.

Table S3. Statistical and quality evaluation of the transcriptomic data.

Table S4. Differentially expressed genes (DEGs) in WP2ΔSP1 by compared with WP2 when using D-AAs as the sole carbon source.

Table S5. Statistical of KEGG enrichment analysis of differentially expressed genes.

Table S6. Gene set enrichment analysis (GSEA) of the transcriptomic data.

Table S7. Taxonomic classification of SP1 and SP1-like viruses (SP1LVs).

Table S8. The sequences of RT-qPCR primers used in this study.

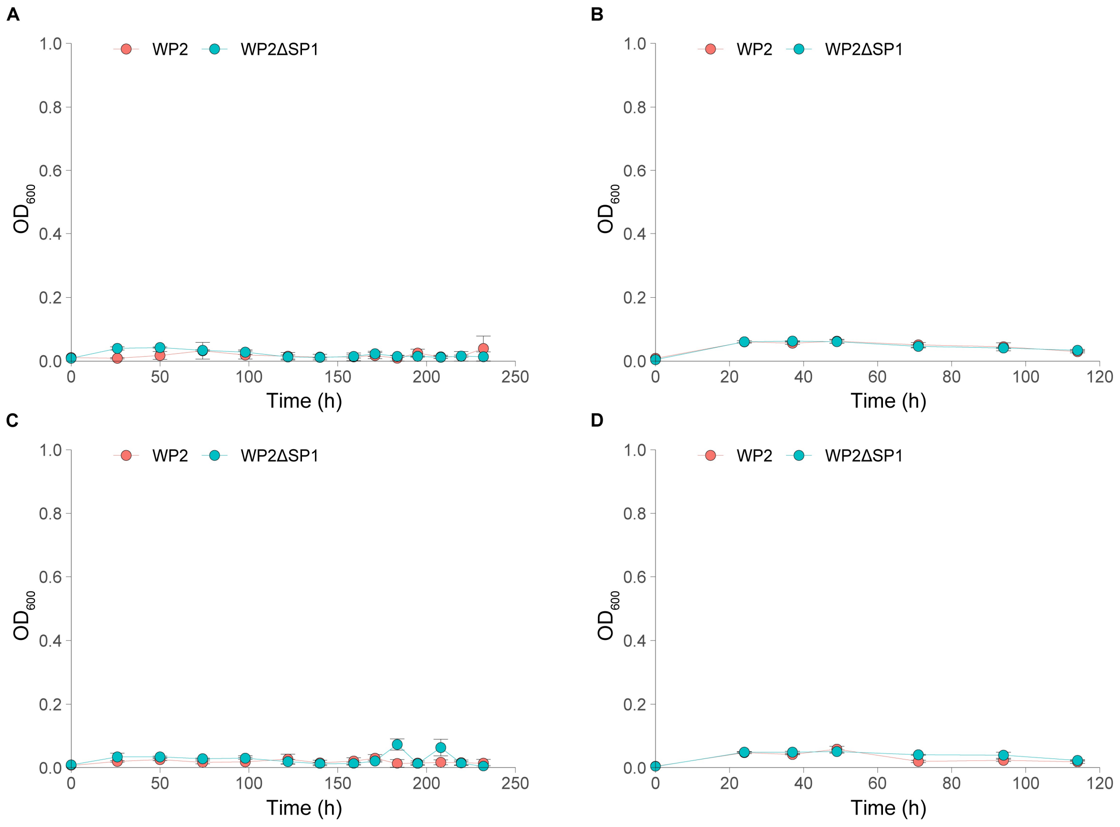

Figure S1. The growth curve of WP2 and WP2ΔSP1 with D-AAs as the sole carbon source. Specifically, the strains were cultured in modified LMO-812 medium with (A) D-Ala, (B) D-Glu, (C) D-Ser, (D) D-Asp as the sole carbon source, respectively. The growth of the strains was detected at optical density at 600 nm (OD_{600}). The error bars indicate standard deviation which were based on three biologically independent samples.

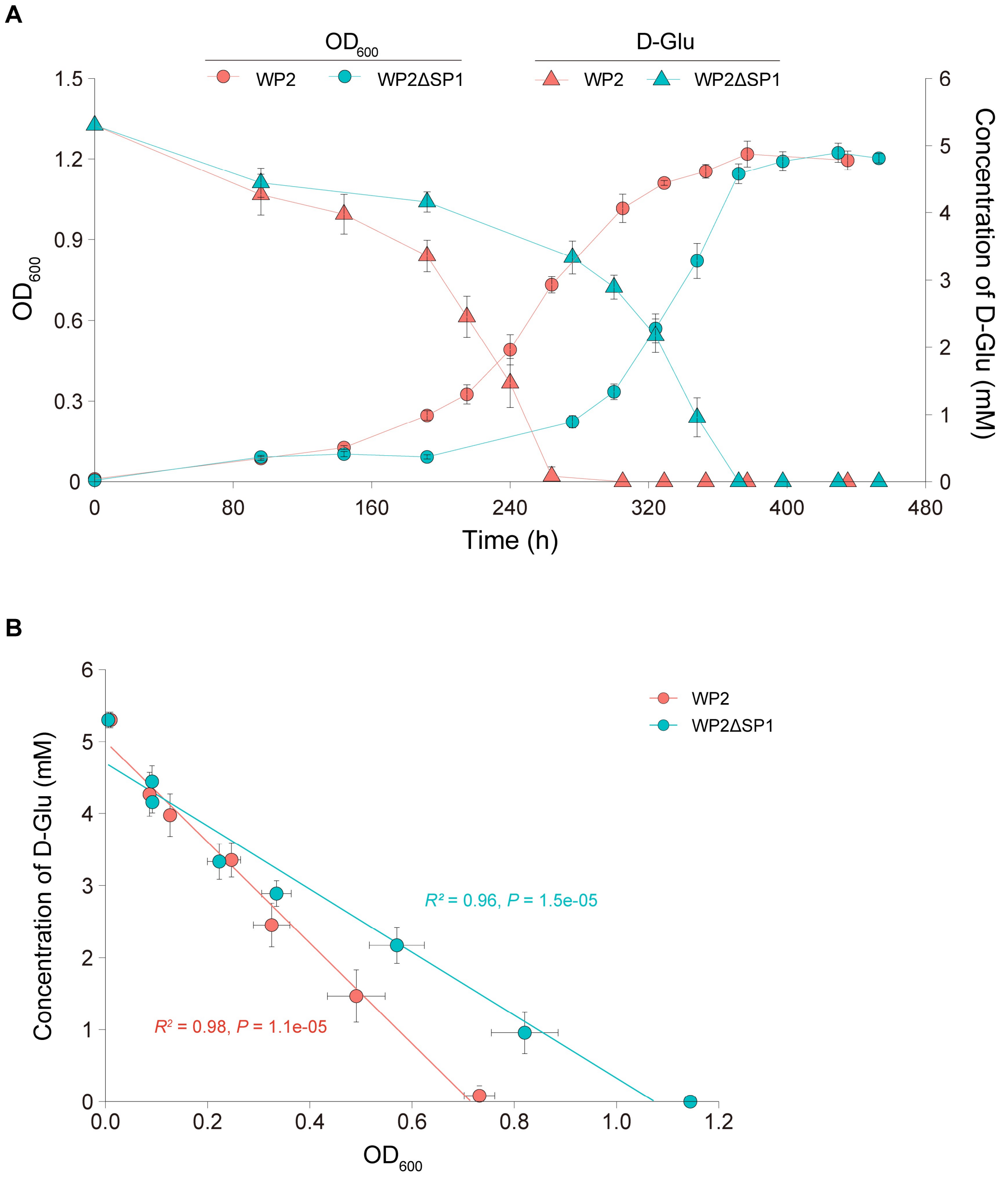

Figure S2. The growth of WP2 and WP2ΔSP1 at 4°C are significantly correlated with the D-Glu degradation. (A) The dynamic change of D-Glu concentration of WP2 and WP2ΔSP1 over the growth phases at 4°C. The strains were cultured in modified LMO-812 medium with D-Glu as the sole nitrogen source. The data shown represent two independent experiments, and the error bars indicate standard deviation which were based on three biologically independent samples. (B) Correlation analysis between D-Glu concentration and growth of WP2 and WP2ΔSP1. The correlation efficiency (R^2) and *P* values of linear regressions are shown for each sub-plot.

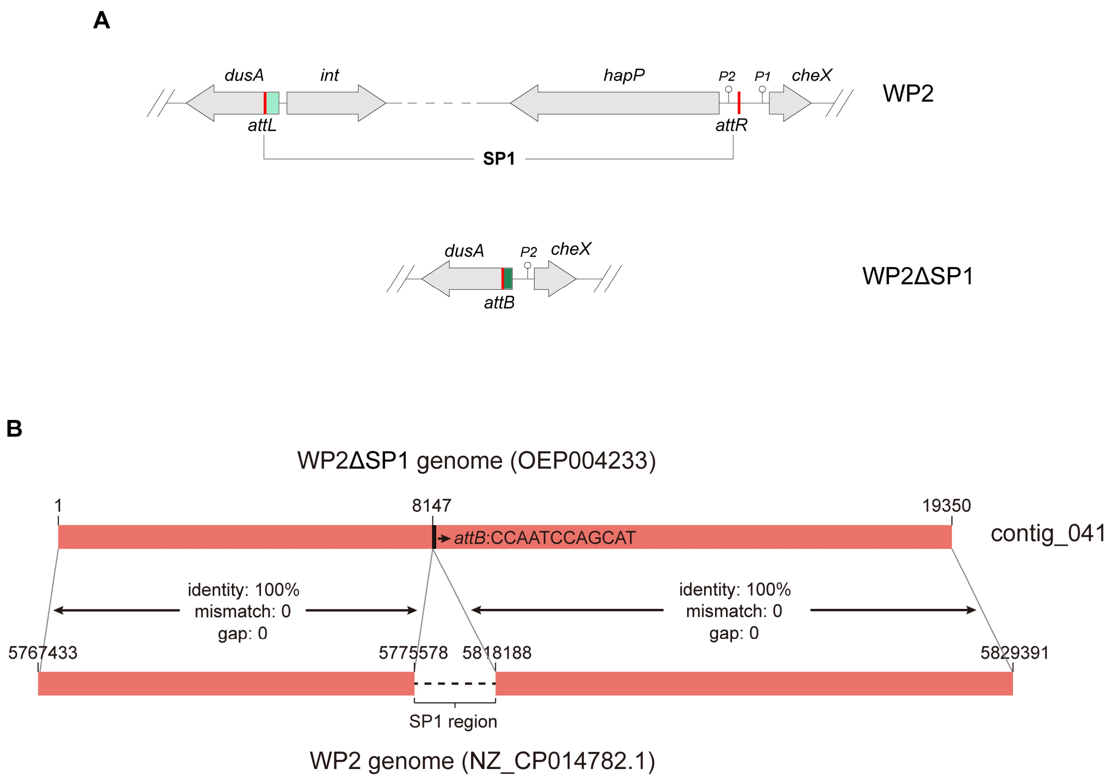

Figure S3. Genomic comparison of WP2ΔSP1 and WP2. (A) Schematic diagram of the knockout of prophage SP1. (B) Schematic diagram of genome sequence alignment between WP2ΔSP1 and WP2. The WP2 genome sequence was retrieved from GenBank database with the accession no. NZ_CP014782.1.

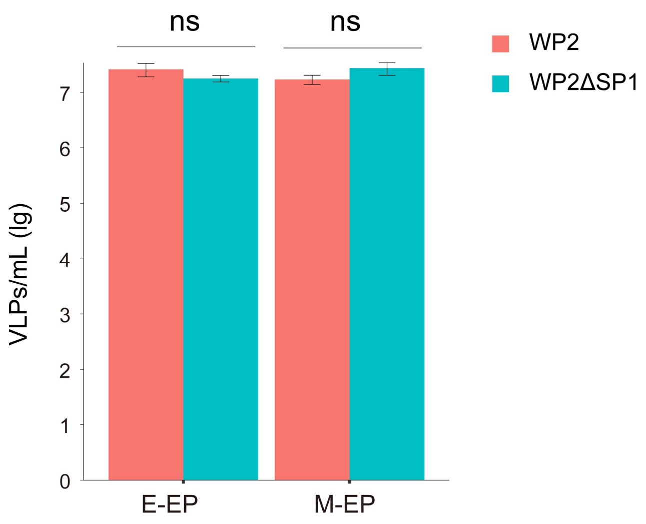

Figure S4. The quantification of virus-like particles (VLPs) produced by WP2 and WP2ΔSP1 at the early (E-EP) and middle (M-EP) stage of exponential growth phase. The data shown represent two independent experiments, and the error bars indicate the standard deviation, which were based on three biologically independent samples. The significances were analysed by two-sided unpaired Student's t test. ns, not significantly different.

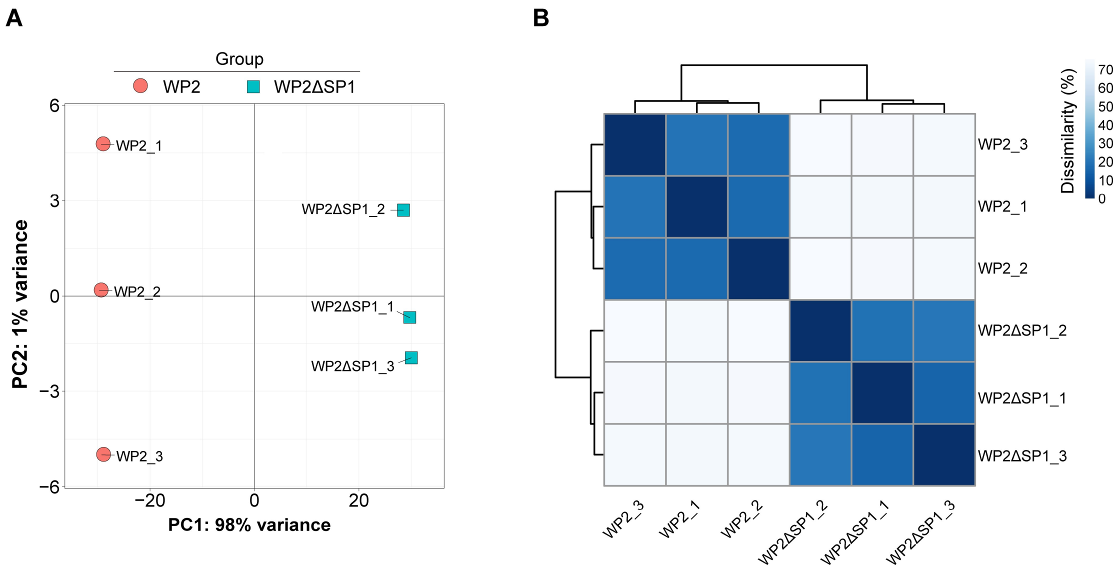

Figure S5. The principal-component analysis (A) and clustering analysis (B) of the six biologically independent samples. Both analyses were based on the expression abundance of genes in these samples belonging to the two strains.

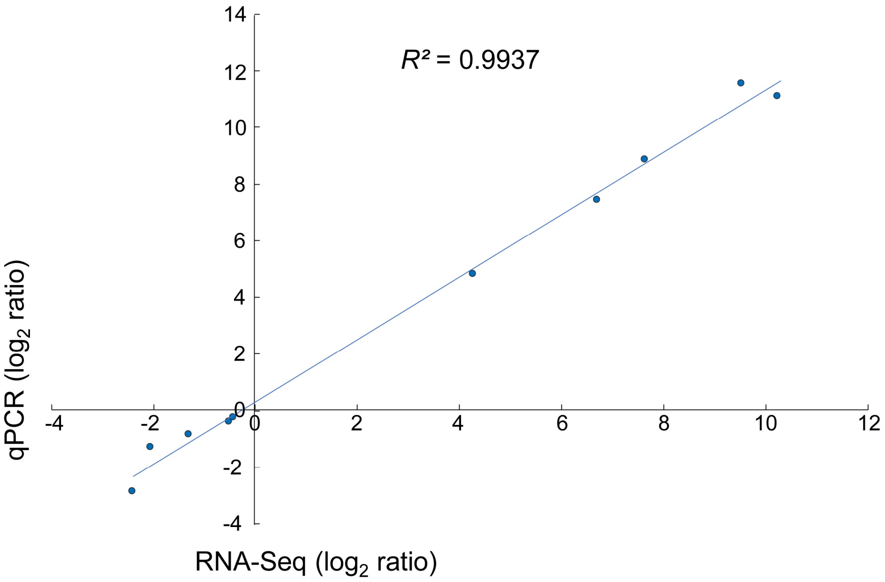

Figure S6. Correlation analysis of the RNA-seq and RT-qPCR assays. Ten genes showing different expression levels were selected randomly for this assay. The RT-qPCR log_2 values were plotted against the RNA-seq log_2 values, and the correlation coefficient (R^2) is shown in the plot.

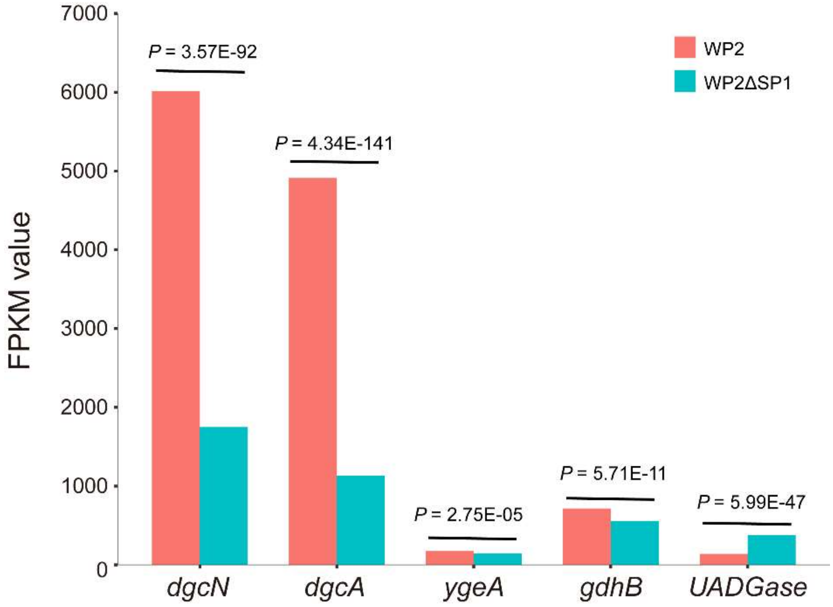

Figure S7. Transcription levels of key genes in D-Glu degradation pathways in WP2ΔSP1 and WP2. FPKM, Fragments Per Kilobase of transcript per Million mapped reads.

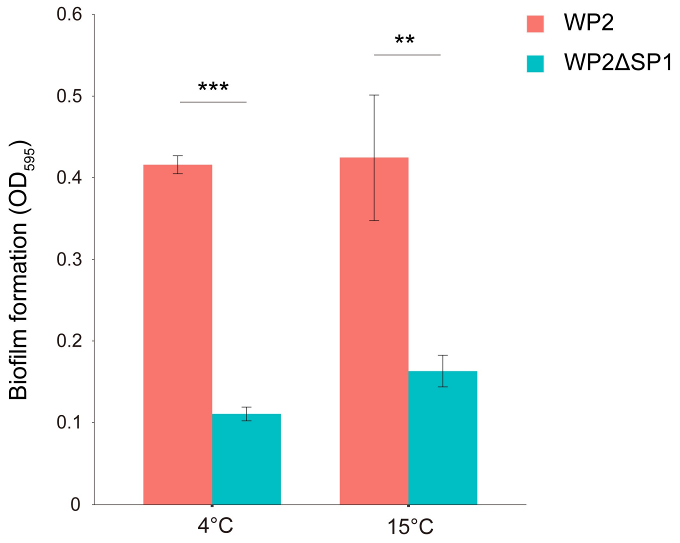

Figure S8. The biofilm formation of WP2 and WP2ΔSP1 at 4°C and 15°C. The data shown represent two independent experiments, and the error bars indicate the standard deviation, which were based on three biologically independent samples. The significances were analysed by two-sided unpaired Student's *t* test. ***, $P < 0.001$; **, $P < 0.01$.

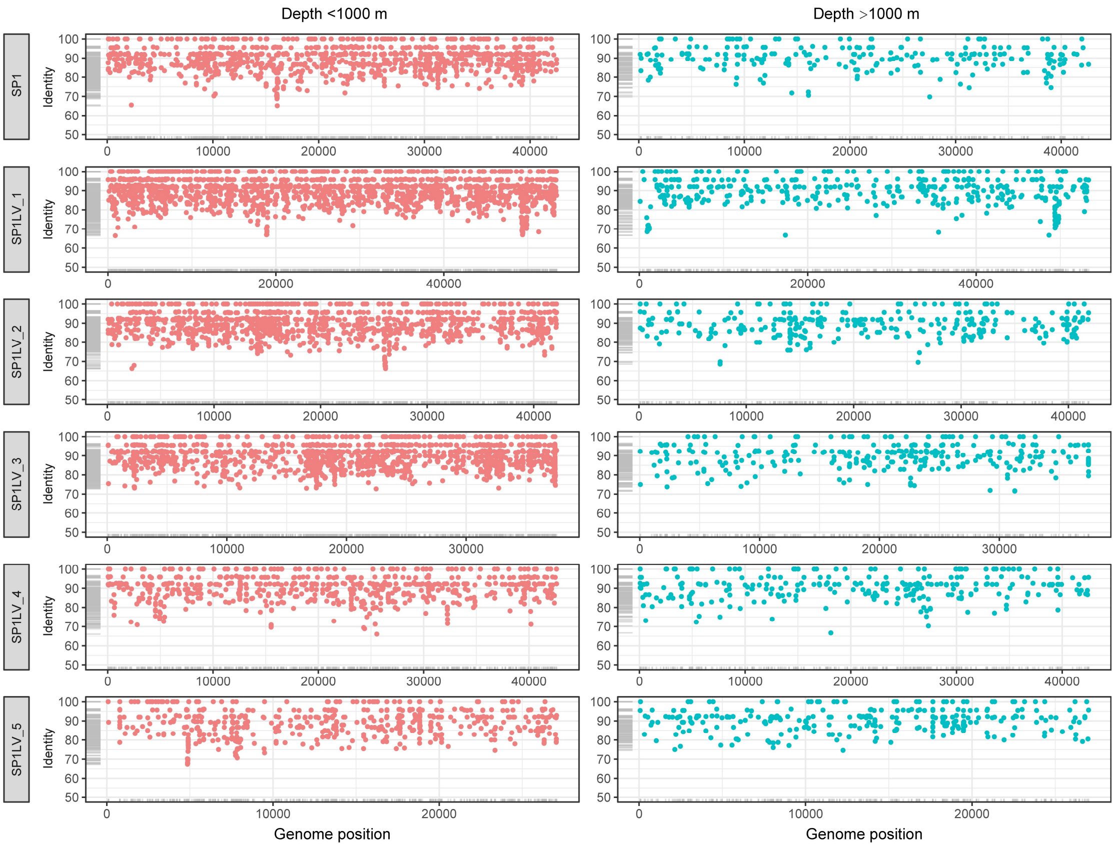

Figure S9. Recruitment analysis of SP1 and SP1-like viruses in the Pacific Ocean Viromes (POV). Recruitment plots are computed using metagenomic raw reads from the POV with water depths <1000 m and ≥1000 m. The recruitment analyses are performed by BLASTn with an e-value cut-off of ≤ 10^{-3}. Only reads that hit with ≥50% identity are shown.

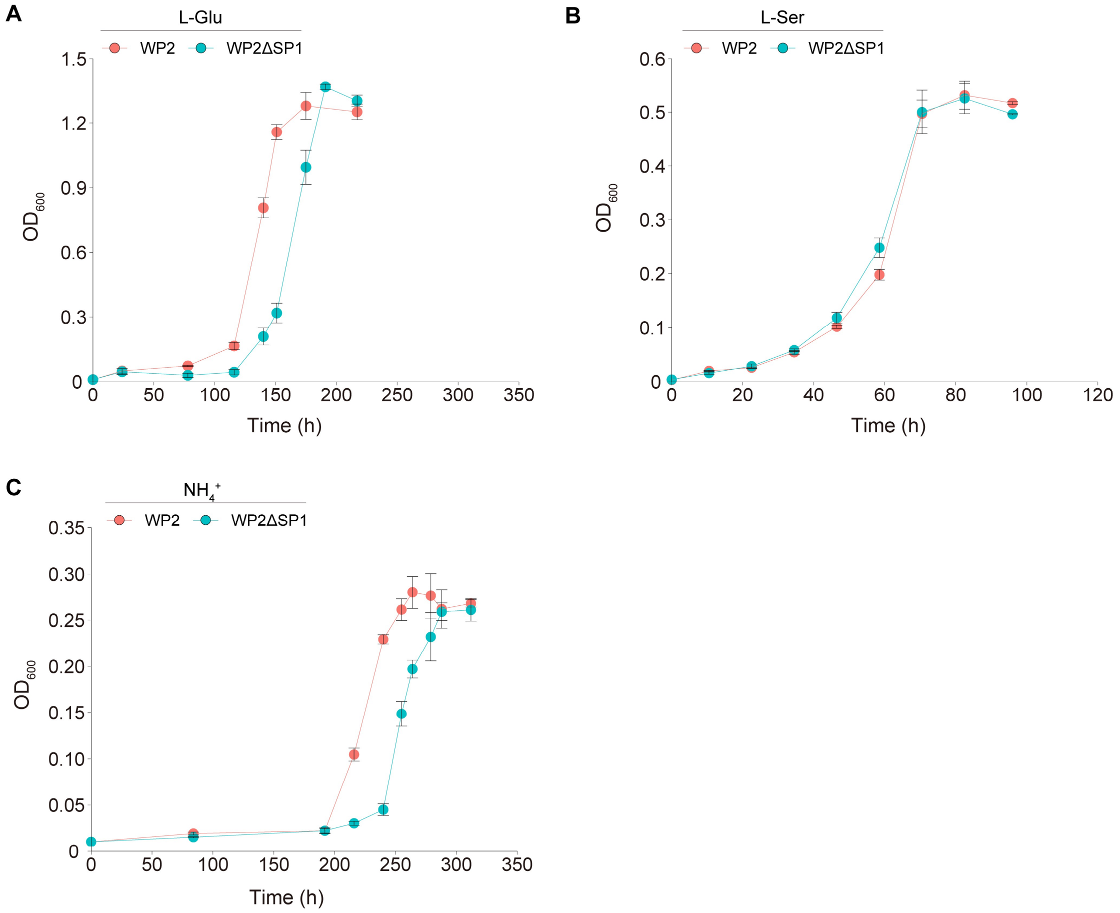

Figure S10. The growth curve of WP2 and WP2ΔSP1 with L-Glu (A), L-Ser (B) and $NH4^+$ (C) as the nitrogen source. The data shown represent two independent experiments, and the error bars indicate the standard deviation, which were based on three biologically independent samples.

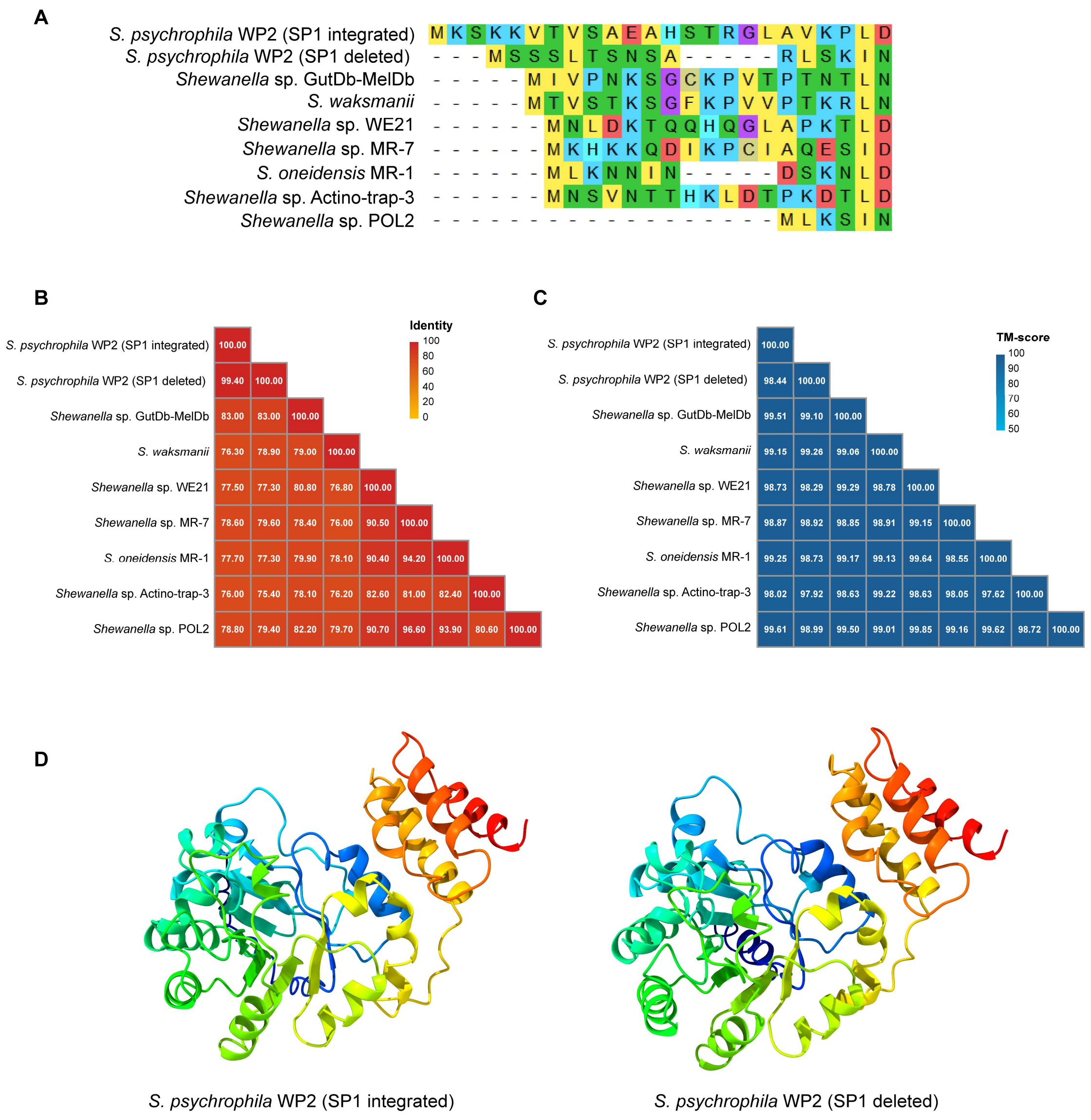

Figure S11. Comparison of DusA protein variants of WP2. (A) Multiple alignment of the amino acid sequences the N-terminal of DusA proteins. (B) Amino acid similarity comparison of DusA proteins. (C) Structural similarity comparison of DusA proteins. The similarity was evaluated based on the TM-score which are indicated by the colour scale. (D) Predicted 3D structures of DusA protein variants of WP2.

Table S1. The genes related to the D-amino acids metabolism in *S. psychrophila* WP2 genome.

Gene ID	Protein ID	Genome coordinates	Amino acids (aa)	Annotation
Sps_05435	WP_077755280.1	6112832..6113698	288	Glutamate racemase
Sps_05173	WP_077755058.1	5820914..5821990	358	Alanine racemase
Sps_01536	WP_077751977.1	1691254..1691949	231	Aspartate/glutamate racemase
Sps_03404	WP_077753560.1	3915968..3917008	346	Proline racemase
Sps_04143	WP_077754190.1	4710067..4711395	442	D-serine ammonia-lyase
Sps_03974	WP_077754049.1	4535379..4536398	339	D-cysteine desulfhydrase
Sps_02107	WP_077752454.1	2379452..2380513	353	N-acetyltransferase DgcN
Sps_02108	WP_077752455.1	2380510..2381577	355	dipeptide epimerase DgcA

Table S3. Statistical and quality evaluation of the transcriptomic data.

Sample	Clean paired reads	Clean reads bases (G)	Q20 (%)	Q30 (%)	GC (%)	Clean data ratio (%)
WP2_1	8,211,557	2.33	99.02	96.03	46.16	89.89
WP2_2	6,300,710	1.78	98.97	95.85	46.39	89.03
WP2_3	9,063,949	2.57	98.96	95.89	46.38	89.63
WP2ΔSP1_1	7,705,912	2.19	98.94	95.85	46.93	89.30
WP2ΔSP1_2	9,386,688	2.66	98.98	96.02	47.46	89.45
WP2ΔSP1_3	8,530,119	2.42	99.00	96.06	47.05	89.39

Table S7. Taxonomic classification of SP1 and SP1-like viruses (SP1LVs).

Virus	AAI (%) (vs SP1)	Gene sharing (%) (vs SP1)	VC Status	VC	Classification
**SP1LV_1**	58.57857143	49.12280702	Clustered	VC_232_0	same genus with SP1
**SP1LV_2**	58.68846154	45.61403509	Clustered	VC_232_0	same genus with SP1
**SP1LV_3**	61.10555556	34.61538462	Clustered	VC_232_0	same genus with SP1
**SP1LV_4**	46.03333333	10.52631579	Clustered	VC_219_0	same family with SP1
**SP1LV_5**	47.11666667	19.35483871	Outlier	-	same family with SP1
**SP1**	-	-	Clustered	VC_232_0	-

Table S8. The sequences of RT-qPCR primers used in this study.

Gene	Sense Primer (5'-3')	Anti-sense Primer (5'-3')
sps_RS02915	CTGCGTCATCGTAATCGTAGTCACA	TGCCTTCAACTTCTGCTTCAACCATA
sps_RS26735	TTGCGGCACGAGTATTAACCTTGA	CCTGTTCACATCTCTGTATTCATCATCTTG
sps_RS07895	CACTGGACACGACTGCTGATAAGATAA	CGGAGGAAACAATGGCGAAGGAA
sps_RS15460	CGCAATTCATTCAGAGCCGCAAT	CACACCTTGGAGCAGAGACACATT
sps_RS13785	AAGTGTAGAGCAAGCAGAGCCATTATC	CGTGACAGCAGTATCTAAGCCTTCC
sps_RS22570	GCTGGATTGGTGGCGATTACTGC	AAGGCACTAGCATCAGACCGAAGAA
sps_RS07785	AATTGATGCTGGCAGTCTGGATAACG	CCTGACTTCGCTGACTCTGGTGTAA
sps_RS16590	CAACAGCAAGCCGCACAGTATCTA	GGCTGCTCCTCTAAGTCCACCTAA
sps_RS07800	CTGTGGTGGTTGTAGTGCCTTGG	CTTCTGAGTCTCTAATCCTTGCTCTTCC
sps_RS28380	TGGCACAGCGTATCGTCAATCG	GTTTCTGGGTTAGCTTGTTGATCTCTGA
SP1-RT	GCAGACATTATTCGAGCTAG	GTCAATCATGTCATCATCCC
rho	GGTTTCCTTCGTAGCGGTGA	TGACGACTGGCTGGTTCATC
